# The architecture of abnormal reward behaviour in dementia: multimodal hedonic phenotypes and brain substrate

**DOI:** 10.1093/braincomms/fcad027

**Published:** 2023-02-09

**Authors:** Anthipa Chokesuwattanaskul, Harmony Jiang, Rebecca L Bond, Daniel A Jimenez, Lucy L Russell, Harri Sivasathiaseelan, Jeremy C S Johnson, Elia Benhamou, Jennifer L Agustus, Janneke E P van Leeuwen, Peerapat Chokesuwattanaskul, Chris J D Hardy, Charles R Marshall, Jonathan D Rohrer, Jason D Warren

**Affiliations:** Dementia Research Centre, Department of Neurodegenerative Disease, UCL Queen Square Institute of Neurology, University College London, London, UK; Division of Neurology, Department of Internal Medicine, King Chulalongkorn Memorial Hospital, Thai Red Cross Society, Bangkok, Thailand; Cognitive Clinical and Computational Neuroscience Research Unit, Faculty of Medicine, Chulalongkorn University, Bangkok, Thailand; Dementia Research Centre, Department of Neurodegenerative Disease, UCL Queen Square Institute of Neurology, University College London, London, UK; Dementia Research Centre, Department of Neurodegenerative Disease, UCL Queen Square Institute of Neurology, University College London, London, UK; Dementia Research Centre, Department of Neurodegenerative Disease, UCL Queen Square Institute of Neurology, University College London, London, UK; Department of Neurological Sciences, Faculty of Medicine, University of Chile, Santiago, Chile; Dementia Research Centre, Department of Neurodegenerative Disease, UCL Queen Square Institute of Neurology, University College London, London, UK; Dementia Research Centre, Department of Neurodegenerative Disease, UCL Queen Square Institute of Neurology, University College London, London, UK; Dementia Research Centre, Department of Neurodegenerative Disease, UCL Queen Square Institute of Neurology, University College London, London, UK; Dementia Research Centre, Department of Neurodegenerative Disease, UCL Queen Square Institute of Neurology, University College London, London, UK; Dementia Research Centre, Department of Neurodegenerative Disease, UCL Queen Square Institute of Neurology, University College London, London, UK; Dementia Research Centre, Department of Neurodegenerative Disease, UCL Queen Square Institute of Neurology, University College London, London, UK; Faculty of Law, Chulalongkorn University, Bangkok, Thailand; Dementia Research Centre, Department of Neurodegenerative Disease, UCL Queen Square Institute of Neurology, University College London, London, UK; Dementia Research Centre, Department of Neurodegenerative Disease, UCL Queen Square Institute of Neurology, University College London, London, UK; Preventive Neurology Unit, Wolfson Institute of Population Health, Queen Mary University of London, London, UK; Dementia Research Centre, Department of Neurodegenerative Disease, UCL Queen Square Institute of Neurology, University College London, London, UK; Dementia Research Centre, Department of Neurodegenerative Disease, UCL Queen Square Institute of Neurology, University College London, London, UK

**Keywords:** reward, frontotemporal dementia, semantic dementia, primary progressive aphasia, Alzheimer’s disease

## Abstract

Abnormal reward processing is a hallmark of neurodegenerative diseases, most strikingly in frontotemporal dementia. However, the phenotypic repertoire and neuroanatomical substrates of abnormal reward behaviour in these diseases remain incompletely characterized and poorly understood. Here we addressed these issues in a large, intensively phenotyped patient cohort representing all major syndromes of sporadic frontotemporal dementia and Alzheimer’s disease. We studied 27 patients with behavioural variant frontotemporal dementia, 58 with primary progressive aphasia (22 semantic variant, 24 non-fluent/agrammatic variant and 12 logopenic) and 34 with typical amnestic Alzheimer’s disease, in relation to 42 healthy older individuals. Changes in behavioural responsiveness were assessed for canonical primary rewards (appetite, sweet tooth, sexual activity) and non-primary rewards (music, religion, art, colours), using a semi-structured survey completed by patients’ primary caregivers. Changes in more general socio-emotional behaviours were also recorded. We applied multiple correspondence analysis and *k*-means clustering to map relationships between hedonic domains and extract core factors defining aberrant hedonic phenotypes. Neuroanatomical associations were assessed using voxel-based morphometry of brain MRI images across the combined patient cohort. Altered (increased and/or decreased) reward responsiveness was exhibited by most patients in the behavioural and semantic variants of frontotemporal dementia and around two-thirds of patients in other dementia groups, significantly (*P* < 0.05) more frequently than in healthy controls. While food-directed changes were most prevalent across the patient cohort, behavioural changes directed toward non-primary rewards occurred significantly more frequently (*P* < 0.05) in the behavioural and semantic variants of frontotemporal dementia than in other patient groups. Hedonic behavioural changes across the patient cohort were underpinned by two principal factors: a ‘gating’ factor determining the emergence of altered reward behaviour and a ‘modulatory’ factor determining how that behaviour is directed. These factors were expressed jointly in a set of four core, trans-diagnostic and multimodal hedonic phenotypes: ‘reward-seeking’, ‘reward-restricted’, ‘eating-predominant’ and ‘control-like’—variably represented across the cohort and associated with more pervasive socio-emotional behavioural abnormalities. The principal gating factor was associated (*P* < 0.05 after correction for multiple voxel-wise comparisons over the whole brain) with a common profile of grey matter atrophy in anterior cingulate, bilateral temporal poles, right middle frontal and fusiform gyri: the cortical circuitry that mediates behavioural salience and semantic and affective appraisal of sensory stimuli. Our findings define a multi-domain phenotypic architecture for aberrant reward behaviours in major dementias, with novel implications for the neurobiological understanding and clinical management of these diseases.

## Teaser

Chokesuwattanaskul *et al*. define four trans-diagnostic, multimodal phenotypes of reward behaviour in Alzheimer’s and frontotemporal dementias. Two core factors drive the emergence and direction of behaviours, with a neural substrate in frontotemporal circuitry that codes the salience and semantic value of sensory objects.

## Graphical Abstract

**Graphical abstract:**
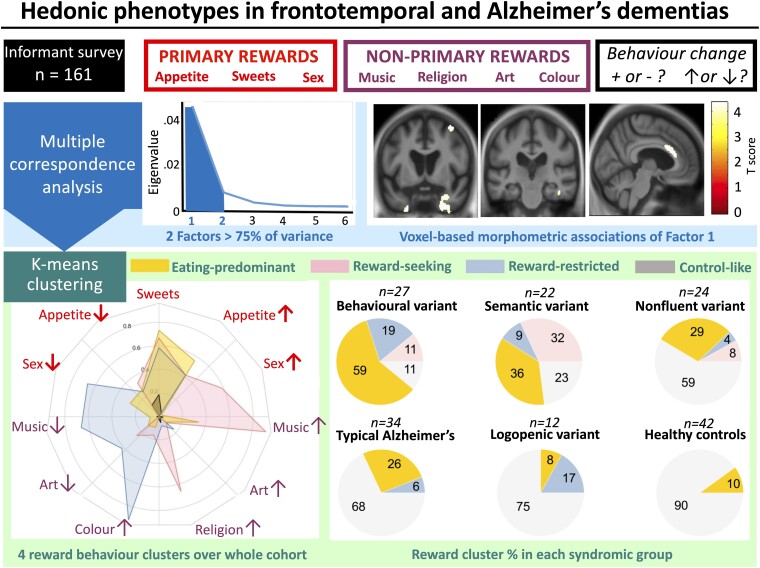

See Horne and Irish (https://doi.org/10.1093/braincomms/fcad045) for a scientific commentary on this article.

## Introduction

Assignment of hedonic value to sensory stimuli is a fundamental task of the brain and plays an essential role in learning from experience, setting behavioural goals and guiding actions that ultimately determine the biological fitness of the individual.^1^ Much human emotional and social behaviour is (explicitly or implicitly) directed toward maximizing rewards while avoiding punishment. ‘Reward’ in its neuropsychological sense encompasses any stimulus or phenomenon that can become the focus of appetitive behaviour and operant conditioning. Primary (or intrinsic) rewards have inherent hedonic or biological value (examples include sweet foods, certain drugs, sex and prosocial intimacy), whereas secondary (extrinsic or non-primary) rewards are generally held to acquire value by association with primary rewards, learning or acculturation (examples include money and music). Impaired reward processing contributes importantly to socio-emotional dysfunction in neurodegenerative diseases.^2^ Abnormalities of appetitive and hedonic behaviours are core to frontotemporal dementia (FTD)^3^ and might constitute an essential pathophysiological principle for understanding and assessing the complex clinical manifestations of these diseases.^2,4–7^ However, the phenotypic spectrum and underlying mechanisms of reward processing in the major dementias remain incompletely defined and poorly understood.

Perhaps the most widely recognized instance of abnormal reward processing in these diseases is altered dietary preference, notably pathological sweet tooth,^8–12^ enshrined in the consensus diagnostic criteria for the behavioural variant of FTD (bvFTD).^13^ However, the objects of hedonic behavioural change in bvFTD span the gamut of primary and non-primary rewards, including alcohol and other drugs,^14^ sex,^12,15,16^ money,^14^ music and environmental sounds,^17,18^ religious experience,^19–21^ art,^22–24^ colours^19^ and various phobic objects.^25^ Clinical experience suggests that altered responsiveness to these objects is hedonically charged—patients with colour obsessions, for instance, commonly express intense liking for particular colours and derive evident pleasure from them. Reward anticipation, appraisal, seeking, satiety and/or conditioning may be affected,^5,6,26,27^ and may be coupled with abnormal interoceptive and/or exteroceptive reactivity.^5,27,28^ It has been proposed that patients with bvFTD may have heightened sensitivity and/or blunted satiety responses to primary rewards, coupled with insensitivity to adverse outcomes.^2,4–6,11,29–31^ However, dichotomized (both seeking and avoidance) responses directed toward food, sex, interpersonal affection, music and other hedonic stimuli have been described within the bvFTD population.^15,18,32,33^

Impaired reward processing is also an important feature in other dementia syndromes. The semantic variant of primary progressive aphasia (svPPA), associated with focal atrophy of the left anterior temporal lobe and its connections, produces behavioural changes broadly similar to bvFTD, frequently including prominent shifts in food preferences, reduced libido, musicophilia and numerophilia.^8,17,18,34–37^ In the right temporal lobe, analogue syndrome of svPPA altered reward behaviour is often particularly striking and extends to hyper-religiosity and increased interest in puzzles and colours.^19–21,38^ Limited information is available concerning reward processing in other primary progressive aphasia (PPA) syndromes; however, patients with the non-fluent/agrammatic variant (nfvPPA) have been reported as behaving similarly to healthy older individuals.^6,18^ Findings in Alzheimer’s disease (AD) have been variable and generally milder than in FTD, but include reduced appetite and less frequent sweet tooth, reduced libido, aversion to environmental sounds (despite retained reactivity to music) and less inclination to gamble,^2,4,6,18,32,39^ perhaps signalling increased sensitivity to satiety and aversive sensory phenomena. On the other hand, patients with AD tend also to retain sensitivity to positive interpersonal reinforcers, in line with their typically preserved social awareness.^14,37^

The brain networks targeted by neurodegenerative pathologies extensively overlap the neural substrates for reward processing implicated in neuroimaging studies in the healthy brain. Reward is mediated by a distributed set of brain regions anchored in ventral striatal, tegmental and basal forebrain structures in the mesolimbic dopaminergic circuit and projecting to prefrontal areas involved in processing stimulus salience, behavioural relevance and affective tone, and anterior temporal structures that code stimulus emotional value and semantic associations.^12,40,41^ The network signatures of bvFTD and svPPA principally involve major hubs of the cingulo-insular ‘salience network’ and the anterior temporal semantic appraisal network, respectively; both syndromes impact the mesolimbic reward circuit.^10,31,42–45^ In AD, primary targeting of the temporo-parietal so-called ‘default mode network’ might shift the balance of activity in connected cerebral networks and, most pertinently, lead to over-activation of the salience network.^14,46^ Voxel-based morphometric (VBM) studies in dementia cohorts have demonstrated grey matter correlates of reward behavioural deficits in striatal, prefrontal, orbitofrontal, anterior and mesial temporal regions.^5,6,10,14^

The complexity of reward behaviours in these diseases raises several fundamental questions. Whereas behavioural changes directed toward primary rewards (notably food and sexual activity) have been reported fairly widely in FTD and AD, behaviours directed toward non-primary rewards appear to be more variable: how do these different kinds of reward behaviour relate to one another, and to different dementia syndromes and pathologies? Do they share common neural substrates? Relatedly, individual patients carrying a particular syndromic diagnosis may exhibit increased or decreased responsiveness to the same hedonic category: what is the significance of this variability, and what are its diagnostic and neurobiological drivers? Disinhibition, obsessionality, apathy, anhedonia and other abnormal socio-emotional behaviours are prevalent in FTD syndromes and likely to impact reward-seeking as well as reward-derived pleasure:^3,8,12,13,47–49^ how and to what extent are hedonic changes linked to more pervasive alterations in socio-emotional behaviour?

Here, we addressed these issues in a survey of behavioural changes in response to a representative sample of primary and non-primary rewards, exhibited by well-characterized patients with bvFTD, all major PPA syndromes and typical AD. Patients were assessed in relation to healthy older people. Survey responses were grounded in the impressions of each patient’s primary caregiver: the usual clinical scenario for recording behavioural changes in daily life. However, our objective in this study was to move beyond clinical observation, to decipher the core phenotypic architecture of reward behaviour in FTD and AD. To this end, we applied unsupervised, unbiased techniques to extract principal factors and hedonic phenotypes across the patient cohort. We further assessed the clinical associations of these core reward parameters with syndromic diagnosis and more general socio-emotional behaviours, and their structural neuroanatomical associations using VBM. Based on previous evidence, we hypothesized that abnormal hedonic behaviours would encompass both primary and non-primary rewards and would be more frequent, salient and diverse in bvFTD and svPPA than in other dementia syndromes. We further hypothesized, based on the dichotomy of hedonic responses reported in these and other dementia syndromes, that their clinical heterogeneity would be underpinned by separable behavioural axes governing the emergence and the direction of hedonic behavioural changes. Finally, we hypothesized that atrophy involving the distributed frontotemporal and striatal circuitry previously implicated in regulating reward processing would constitute an essential neuroanatomical substrate for these behavioural changes.

## Materials and methods

### Participants

The patient cohort was recruited via a specialist cognitive disorders clinic and comprised 27 patients with bvFTD, 22 with svPPA, 24 with nfvPPA, 12 with logopenic variant primary progressive aphasia (lvPPA) and 34 with typical amnestic AD (henceforth AD). All patients fulfilled consensus criteria for the relevant diagnosis,^13,50,51^ with supportive general neuropsychological features, compatible brain MRI findings and mild to moderately severe disease. No patients with pathogenic genetic mutations were included. All 15 patients with clinically typical AD who underwent lumbar puncture and/or brain amyloid-positron emission tomography had biomarker profiles consistent with underlying AD pathology, based on local criteria. Each patient had a primary caregiver who was able to supply information about their behaviour currently and premorbidly. Forty-two healthy older participants with no history of cognitive complaints or psychiatric illness were recruited via the Dementia Research Centre control database. All participants were native British residents with a similar socio-cultural background. Demographic and general clinical characteristics of the participant groups are summarized in Table 1; details of their neuropsychological profiles are provided in Supplementary Table 1.

**Table 1:** Demographic and clinical characteristics and prevalence of reward behavioural changes for all participant groups

Characteristic	Controls	AD	lvPPA	bvFTD	svPPA	nfvPPA
*General*						
No. (m:f)	42 (23:19)	34 (18:16)	12 (10:2)	27 (20:7)	22 (13:9)	24 (14:10)
Handed (R:L)	40:2	30:4	11:1	26:1	21:1	23:1
Age (y)	66.8 (6.5)	70.7 (8.1)	67.6 (9.1)	66.7 (7.7)	66.3 (7.1)	70.9 (8.1)
Education (y)	16.0 (12.2–17.0)	16.0 (12.2–16.0)	15.0 (13.8–16.2)	14.0 (12.0–16.0)	16.0 (11.2–16.0)	13.5 (11.0–16.0)
Illness (y)	NA	5.3 (4.2–7.6)	5.3 (4.3–6.8)	4.9 (4.0–6.0)	5.3 (4.6–6.3)	4.3 (2.6–5.1)
MMSE score	30.0 (29.0–30.0)	**18.5 (16.2–25.0)**	**12.5 (10.0–17.0)^a,b^**	**24.0 (21.0–27.5)^c^**	**23.5 (18.0–28.5)^c^**	**25.5 (17.2–8.0)**
*Reward domains*						
No. (%)						
Primary						
Any	4 (10)	**20 (59)**	4 (33)	**24 (89)^d,c,e^**	**18 (82)^c^**	**12 (50)^a^**
Inc	2 (5)	**13 (38)^a^**	3 (25)	**23 (85)^d,c,e^**	**15 (68)**	**10 (42)^a^**
Dec	2 (5)	**9 (26)**	2 (17)	**13 (48)**	**8 (36)**	4 (17)
Appetite						
Any	2 (5)	**14 (41)**	1 (8)	**19 (70)^c^**	**14 (64)^c^**	**11 (46)**
Inc	0 (0)	**5 (15)^a^**	0 (0)^a^	**15 (56)^c,d^**	**8** (36)	**7 (29)**
Dec	2 (5)	9 (26)	1 (8)	4 (15)	6 (27)	4 (17)
Sweet tooth						
Any (Inc)	2 (5)	**12 (35)^a^**	3 (25)^a^	**20 (74)^c–e^**	**13 (59)**	**7 (29)^a^**
Sex						
Any	0 (0)	2 (6)^a^	2 (17)	**14 (52)^d,e^**	**6 (27)**	3 (12)^a^
Inc	0 (0)	0 (0)	0 (0)	3 (11)	3 (14)	2 (8)
Dec	0 (0)	2 (6)^a^	2 (17)	**11 (41)^d,e^**	3 (14)	1 (4)^a^
Non-primary						
Any	11 (26)	5 (15)^a,b^	5 (42)^a^	**23 (85)^c–e^**	**17 (77)^d,e^**	8 (33)^a,b^
Inc	9 (21)	4 (12)^a,b^	4 (33)	**20 (74)^d,e^**	**14 (64)^d^**	7 (29)^a^
Dec	2 (5)	3 (9)	3 (25)	7 (26)	7 (32)	2 (8)
Religion						
Any (Inc)	1 (2)	0 (0)	1 (8)	4 (15)	5 (23)	2 (8)
Music						
Any	4 (10)	4 (12)^a,b^	4 (33)	**16 (59)^d,e^**	**13 (59)^d,e^**	2 (8)^a,b^
Inc	3 (7)	2 (6)^a,b^	1 (8)	**12 (44)^d,e^**	**11 (50)^d,e^**	1 (4)^a,b^
Dec	1 (2)	2 (6)	3 (25)	4 (15)	2 (9)	1 (4)
Art						
Any	2 (5)	2 (6)	1 (8)	**9 (33)**	5 (23)	3 (12)
Inc	1 (2)	1 (3)	1 (8)	4 (15)	0 (0)	1 (4)
Dec	1 (2)	1 (3)	0 (0)	5 (19)	5 (23)	2 (8)
Colours						
Any (Inc)	6 (14)	2 (6)	2 (17)	7 (26)	4 (18)	3 (12)
All	13 (31)	**22 (65)^a^**	8 (67)	**26 (96)^d,e^**	**19 (86)**	**16 (67)^a^**

The table summarizes general demographic and clinical data, and the prevalence of altered reward behaviours in each of the sampled hedonic domains for each participant group, as determined from the symptom survey (see text and Supplementary Table 2). Counts, mean (standard deviation) or median (interquartile range) are shown for demographic and clinical data. Raw counts and percentage of group exhibiting each symptom are shown for each reward domain. Significant differences (*P*_FDR_ < 0.05) between patient groups and healthy controls are in bold; significant differences between patient groups are coded as follows: ^a^significantly different from bvFTD, ^b^significantly different from svPPA, ^c^significantly different from lvPPA, ^d^significantly different from AD, ^e^significantly different from nfvPPA. AD, patient group with typical Alzheimer’s disease; Any, any change in responsiveness toward that reward; bvFTD, patient group with behavioural variant frontotemporal dementia; Change, overall frequency and dominant direction of behavioural alteration (see text); Controls, healthy control group; Dec, primarily decreased responsiveness; f, female; Handed, handedness; Illness, estimated symptom duration; Inc, primarily increased responsiveness; symptom duration; L, left; lvPPA, patient group with logopenic variant primary progressive aphasia; m, male; MMSE, Mini-Mental State Examination (maximum score 30);^52^ no., number; nfvPPA, patient group with non-fluent/agrammatic variant primary progressive aphasia; R, right; svPPA, patient group with semantic variant primary progressive aphasia; y, years.

The study was approved by the University College London institutional ethics committee and all participants gave informed consent in accordance with the Declaration of Helsinki.

### Collection of data on hedonic and related socio-emotional behaviours

We used a semi-structured symptom survey (see Supplementary Table 2) to sample behavioural changes representing both primary rewards—changes in appetite, sweet tooth and sexual behaviour—and non-primary rewards—music, religion, art and colours—in a uniform framework. The selection of surveyed hedonic domains was experimenter-driven and necessarily incomplete; it was informed by our cumulative clinical experience as well as available published evidence in the target diseases.^2,3,8,11–13,15,18,19,23^ Food and sex are primary rewards commonly implicated in previous work in FTD and AD, and within the domain of food, changes in food preference (most commonly, sweet tooth) and more generalized changes in appetite appear to be at least partly dissociable. Among diverse candidate non-primary reward stimuli, altered responses to music, religion, visual art and colours are often clinically striking in this disease spectrum and represent ‘abstract’ stimuli that (in variable degree) lack intrinsic biological and/or social value. Certain other candidate reward objects were not included because we considered they would be challenging to capture in the same framework: money, for instance, is difficult to disambiguate hedonically from the commodities it buys (and tends moreover to be regulated by caregivers), while interpersonal affinity is potentially confounded by other cognitive and behavioural factors (patients with AD, for example, often withdraw from social contact due to a dislike of busy auditory environments, while the frequently marked loss of social reactivity in bvFTD might reflect impaired mentalizing rather than hedonic devaluation *per se*).

Changes in reward behaviour within the sampled domains encompassed any observable evidence of altered hedonic responsiveness (liking, enjoyment and/or interest, e.g. seeking or avoidance of the relevant item). We sought to capture both the presence of reward behavioural changes and where relevant, their direction (increased or decreased) compared with the person’s behaviour 10 years previously, an arbitrary interval that predated the onset of symptoms for all patients.

The survey was completed by usual primary caregivers on behalf of each patient and by healthy control participants themselves. If the patient had exhibited evidence of behavioural changes in either direction from time to time, the caregiver was asked to record the dominant direction of the alteration. For sweet tooth, religion and colours, only heightened responsiveness was recorded, as we considered that reduced responsiveness in these domains might be generally less socially or culturally salient and/or difficult to identify accurately. In this first survey, we did not ask caregivers to quantify behaviour changes for frequency or severity, because we reasoned it would be challenging to capture the diverse range of sampled rewards on a common rating scale. However, survey respondents were invited to further describe any behavioural change (recorded as a free text entry). Prior to completing the survey, caregivers were given examples of relevant types of behavioural changes that they might record.

Caregivers were additionally asked about the presence of other, more general changes in patients’ socio-emotional behaviour relevant to abnormal reward processing and/or its expression, following a similar survey protocol. These surveyed behavioural changes comprised: disinhibition (socially inappropriate or impulsive behaviour); apathy (loss of interest, reduced motivation, decreased initiation of activities); ritualistic, compulsive or obsessional behaviour; loss of empathy (diminished responsiveness to other people’s needs or feelings, diminished personal warmth) and inappropriate humour (failing to laugh at things others find amusing or laughing at things others generally do not find amusing).

### Analysis of clinical and behavioural data

#### Statistical analysis

Demographic and clinical data and raw reward symptom prevalence data were analysed using Python (v3.8.5) software. The prevalence of each reward symptom was compared between participant groups.

ANOVA and Kruskal–Wallis’s test were used to compare continuous variables and chi-square tests were used to compare categorical variables across syndromic groups and clusters; Fisher’s exact tests were used when expected counts were small for a categorical variable in each group or cluster. *Post hoc* pair-wise comparisons were carried out when applicable, with a correction for false discovery rate over multiple comparisons. For all tests, a threshold *P* < 0.05 was accepted as the criterion for statistical significance.

#### Multiple correspondence analysis

To identify the underlying core architecture of abnormal reward behaviour in the participant cohort, we applied multiple correspondence analysis (MCA),^53^ a type of factor analysis designed for categorical datasets that make no prior assumptions about the data distribution (further background in Supplementary material).

Categories of reward behaviour were dummy coded according to the survey response type (increased, decreased or no change) and factor extraction was performed across all responses (reward ‘features’). Eigenvalues (principal inertias) and explained variance were calculated for each successive orthogonal factor and a Greenacre correction was implemented to account for eigenvalue inflation resulting from the additional dummy columns. The ‘elbow method’ on the scree plot of eigenvalues (Supplementary Fig. 1) was used to determine the number of factors to retain. The squared cosine was used to quantify the strength of the association of each reward feature with each principal factor. Associations of each principal factor with general disease characteristics and general socio-emotional behaviours were assessed.

#### Cluster analysis

*K*-means clustering, an unsupervised machine learning distance-based algorithm, was used to characterize reward phenotypes across the participant cohort; this algorithm groups together subjects with similar featural profiles while maximizing the difference between clusters. All retained principal factors identified on MCA were entered into the *k*-means clustering model. The appropriate number of clusters was determined using the elbow method on the sum-of-squared-errors plot (see Supplementary Fig. 2). A cluster stability analysis was applied to test the robustness of the model using a bootstrapping approach: briefly, we sampled 80% of the participants from the combined cohort with replacement after each iteration for a total of 5000 iterations and ran the *k*-means clustering model on all samples (further details in the Supplementary material). Over all iterations, the average percentage of iterations on which participants were assigned to the same cluster was reported as the cluster stability index. We assessed any associations of reward behavioural clusters with a diagnostic group, and reported changes in other socio-emotional behaviours (disinhibition, apathy, obsessionality, loss of empathy, inappropriate humour) and general demographic and clinical variables.

### Brain imaging acquisition and analysis

Each patient had a T_1_-weighted brain volume acquired on the same Siemens Prisma 3 T MRI scanner; details of MRI acquisition and pre-processing are in given the Supplementary material.

In separate regression models, we assessed the association of regional grey matter volume (indexed as voxel intensity) with the retained principal factors identified on MCA. Age and total intracranial volume were incorporated as covariates in each model, and interaction with syndromic group membership was also modelled, to take account of variations in atrophy pattern attributable to syndromic diagnosis *per se*. Positive and negative associations with regional grey matter volume were assessed separately for each factor. Statistical parametric maps were generated using an initial uncorrected threshold of *P* < 0.001 and assessed at a peak-level threshold of *P* < 0.05, after family-wise error correction for multiple voxel-wise comparisons over the whole brain.

## Results

### Participant group characteristics and reward behavioural profiles

Participant groups did not differ significantly in age, gender distribution, handedness or years of education; the patient groups did not differ in mean symptom duration, but did differ in overall disease severity [Mini-Mental State Examination (MMSE) score; see Table 1]. General neuropsychological and socio-emotional behavioural data are presented in Supplementary Table 1.

Comparing the relative prevalence of abnormal reward behaviours from the caregiver survey over the combined patient cohort (see Table 1), we found that among those behaviours sampled, the most frequent was the alteration in appetite and/or sweet tooth, each present in around half of all patients. However, over all hedonic domains surveyed, quantitatively and qualitatively distinct profiles were identified in different patient groups (Table 1). The bvFTD group had the highest overall frequency of altered reward behaviours (96%), followed by the svPPA group (86%); the frequency was lower in the nfvPPA, lvPPA and AD groups (each around 66%), but still substantially higher than in healthy controls (31%). Behavioural changes directed toward primary rewards, in particular appetite and/or sweet tooth, were prevalent in each patient group and significantly more frequent than in healthy controls (*P*_FDR_ < 0.05 for all patient groups except lvPPA). Decreased appetite was particularly associated with AD and svPPA, while only patients with bvFTD exhibited significantly diminished sexual behaviour relative to healthy controls (*P*_FDR_ < 0.001). Behavioural changes directed toward non-primary rewards were significantly more prevalent in the bvFTD and svPPA groups than in healthy controls (both *P*_FDR_ < 0.001) and other patient groups (all *P*_FDR_ < 0.05 except svPPA versus lvPPA *P*_FDR_ = 0.12). Music was the non-primary reward domain most commonly affected in both bvFTD and svPPA, followed by art and colours in bvFTD and religiosity in svPPA. Reduced responsiveness to music distinguished the lvPPA group here.

Representative caregiver comments are presented in Supplementary Table 3. These attest that even infrequently observed reward behaviours could be striking (for example, a new inclination to dress entirely monochromatically in some patients with svPPA).

### Principal reward factor characteristics

**Figure 1:**
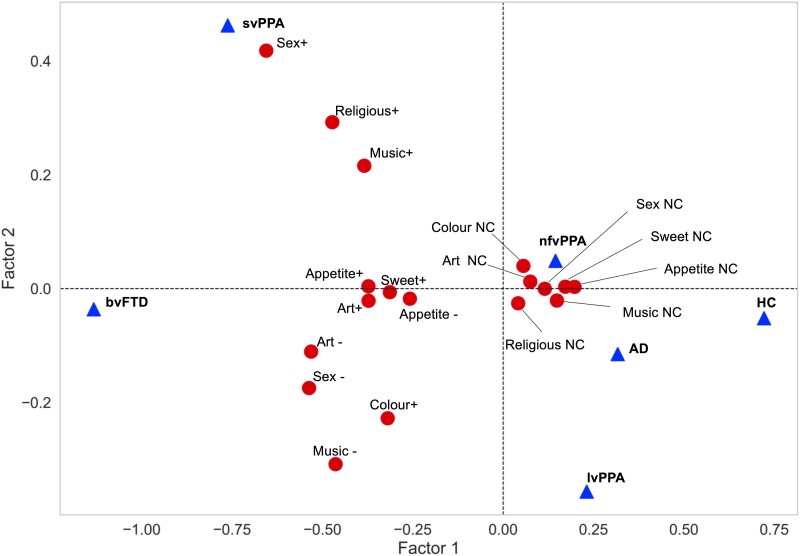
**Principal factors governing reward behavioural changes in the study cohort.** The plot aligns reward behavioural response categories or ‘features’ aligned to principal factors for all 161 participants, based on an MCA of the reward symptom survey. Factor 1 is presented on the *x*-axis and factor 2 on the *y*-axis. The (*x*, *y*) coordinates of each reward survey response category or feature (dots) represent the factor score (in arbitrary units) of that feature for factor 1 and factor 2, respectively (see also Supplementary Table 4). The factor score quantifies the contribution of that feature to the factor. Increasing discrimination between features corresponds to increasing distance along each axis; the greater separation of features along the *x*-axis (note change of scale) indicates that factor 1 accounts for most of the variance in reward behavioural features in the participant cohort, discriminating presence from absence of altered reward behaviours. Altered responsiveness to sex, music, religion and colour are relatively well discriminated by factor 2 (*y*-axis). Reward features that aggregate tend to co-occur in the same participants or group of participants. Features that are more distant from the origin are less frequently reported and signify deviation from average cohort behaviour. Diagnostic group features are visualized in this plot as supplementary variables (triangles); their coordinates were derived by projecting them onto principal factors 1 and 2. The positioning of the bvFTD and HC diagnostic groups at opposite ends of the *x*-axis relates to their strong association with factor 1 (i.e. discriminating presence from absence of altered reward behaviours); the positioning of the svPPA and lvPPA groups at opposite ends of the *y*-axis relates to their strong association with factor 2 (i.e. discriminating the direction of altered reward behaviours). AD, patient group with typical Alzheimer’s disease; Appetite ±, appetite increased/decreased; Art ±, art responsiveness increased/decreased; bvFTD, patient group with behavioural variant frontotemporal dementia; Colour +, increased responsiveness to colours; HC: healthy control group; lvPPA, patient group with logopenic variant primary progressive aphasia; Music ±, music responsiveness increased/decreased; NC, no change; nfvPPA, patient group with non-fluent/agrammatic variant primary progressive aphasia; Religious +, increased religiosity; Sex ±, libido increased/decreased; svPPA, semantic variant primary progressive aphasia; Sweet +, increased sweet tooth.

Based on the scree plot of the MCA of reward behaviour survey responses (Supplementary Fig. 1), we retained the leading two factors: the first factor explained most of the total variance (66.8% after Greenacre correction), whereas the second factor explained most of the remaining variance (an additional 9.8% after Greenacre correction). The relations of reward features and diagnostic groups (considered here as supplementary variables; see the Supplementary material) to each of the two principal factors are plotted in Fig. 1. Factor 1 separated features corresponding to any reward behavioural change from absence of change, whereas factor 2 separated features corresponding mainly to increased reward response (heightened responsiveness to music, sex and religion with altered appetite and sweet tooth) from features corresponding mainly to reduced reward response (diminished responsiveness to sex and music with altered response to art, but also heightened response to colours). Diagnostic groups were differentiated by the two principal factors (Fig. 1): the bvFTD and healthy control groups were maximally separated along factor 1 axis, whereas the svPPA and lvPPA groups were maximally separated along factor 2 axis.

**Figure 2:**
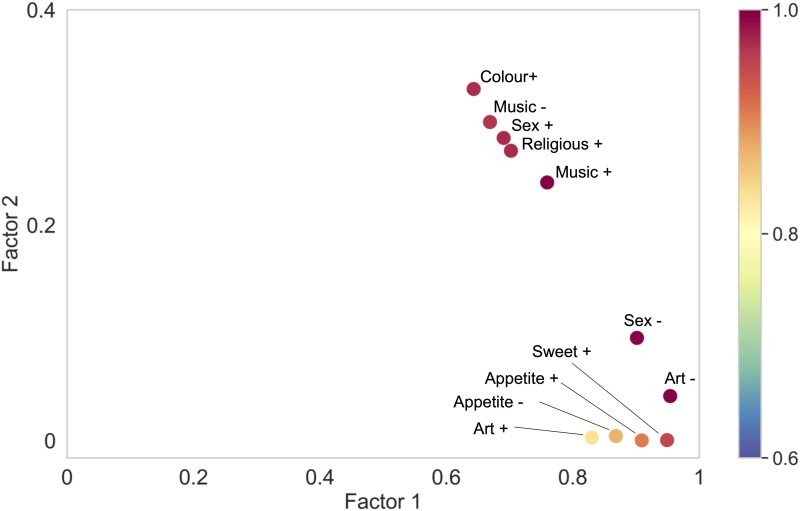
**Correlation of principal reward factors with reward behavioural changes.** The plot shows the squared cosine values of each reward feature with the two principal reward factors, extracted from the MCA (*n* = 161 participants). The (*x*, *y*) coordinates here represent the squared cosines of each reward feature on factor 1 and factor 2, respectively; note change of scale between axes (see also Supplementary Table 4). The bar on the right codes the sum of squared cosines of factor 1 and 2 for each feature. The squared cosine value quantifies how strongly a feature is associated with a particular factor; it is related to distance along the factor axis from the origin in Fig. 1. Features with higher correlation values are better segregated from the ‘average’ feature profile of the study cohort by that factor. Appetite *±*, appetite increased/decreased; Art *±*, art responsiveness increased/decreased; Colour +, increased responsiveness to colours; Music *±*, music responsiveness increased/decreased; Religious +, increased religiosity; Sex *±*, libido increased/decreased; Sweet +, increased sweet tooth.

Reward behaviours were generally well represented by the combination of the two principal factors, as indicated by a sum of squared cosines >0.6 for all features (Fig. 2 and Supplementary Table 4). Sweet tooth, increased appetite, reduced libido and altered responsiveness to art were most strongly correlated with factor 1; heightened responsiveness to colour, religiosity, increased libido and altered responsiveness to music were most strongly correlated with factor 2. Associations of each principal reward factor with general disease characteristics and other socio-emotional behaviours are summarized in Supplementary Table 5. Both factors were significantly associated (*P* < 0.05) with each of the examined general socio-emotional behaviours (disinhibition, apathy, obsessionality, loss of empathy, inappropriate humour); however, neither was significantly associated with overall illness (symptom) duration or disease severity (as indexed by MMSE).

### Definition of reward phenotypic clusters

**Figure 3:**
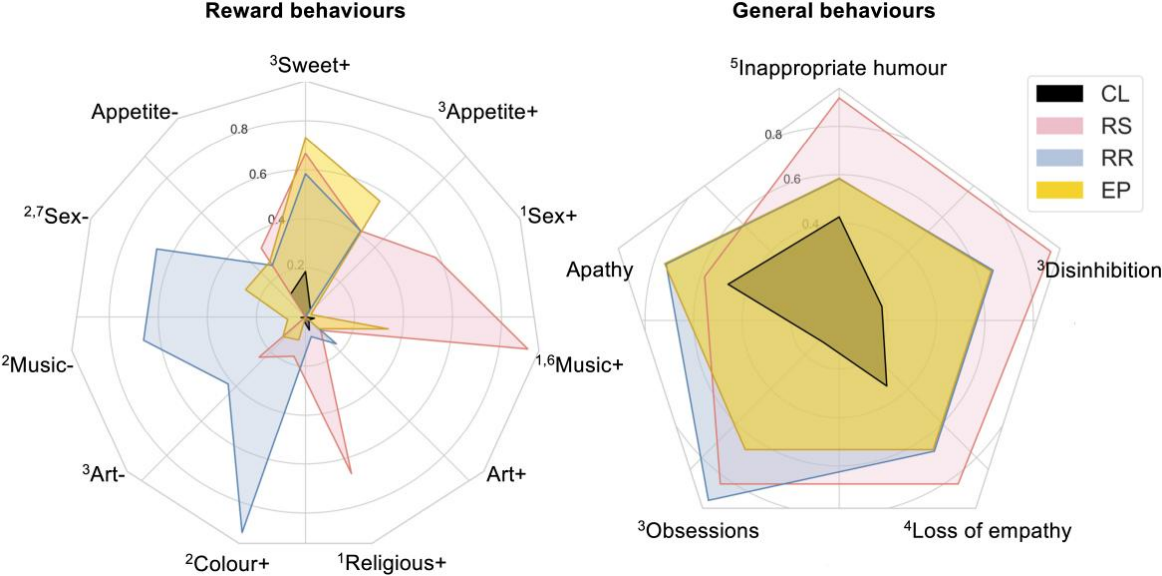
**Characteristics of reward behavioural phenotypic clusters.** Radar plots depict the four reward behavioural phenotypic clusters in the combined patient cohort (*n* = 119 participants), the ‘reward-seeking’ (RS) cluster, the ‘reward-restricted’ (RR) cluster, the ‘eating-predominant’ (EP) cluster and the ‘control-like’ cluster (CL). Behavioural changes of interest are plotted around the circumference; concentric circles represent the proportion of participants exhibiting that change in each cluster (plotted along the radius). The left panel shows the proportion of participants in each cluster with particular reward features; the right panel shows the proportion of participants in each cluster with more general socio-emotional behavioural changes. Pair-wise comparisons between clusters using the chi-square test with *post hoc* correction (*P*_FDR_ < 0.05) are coded as follows: ^1^RS > RR, EP, CL; ^2^RR > RS, EP CL;^3^RS, RR, EP > CL; ^4^RS, EP > CL; ^5^RS > CL; ^6^EP > RR, CL; ^7^EP > CL (see also Supplementary Tables 6 and 7). Appetite ±, appetite increased/decreased; Art ±, art responsiveness increased/decreased; Colour +, increased responsiveness to colours; Music ±, music responsiveness increased/decreased; CL, ‘control-like’ cluster; Religious +, increased religiosity; RR, ‘reward-restricted’ cluster; RS, ‘reward-seeking’ cluster; SC, ‘subtle change’ cluster; Sex ±, libido increased/decreased; Sweet +, increased sweet tooth.

Four clusters of reward behavioural phenotypic features were found to optimally segregate the participant cohort, with a high cluster stability index (97.5%; see Supplementary Figs 2 and 3). We designate these the ‘reward-seeking’ (12 patients), ‘reward-restricted’ (12 patients), ‘eating-predominant’ (41 patients) and ‘control-like’ (54 patients) clusters, based on their behavioural phenotypic features; the demographic, clinical and neuropsychological characteristics of these clusters are summarized in Fig. 3 and Supplementary Tables 6 and 7.

The ‘reward-seeking’ and ‘reward-restricted’ clusters were defined on the basis of differential responsiveness to music, sex, religion and colours. A substantial proportion of patients in the ‘reward-seeking’ cluster exhibited increased responsiveness to music (92%), religion (67%) and/or libido (58%) (all significantly more prevalent compared with the ‘reward-restricted’ and ‘control-like’ clusters, *P*_FDR_ < 0.05). In contrast, a high proportion of patients in the ‘reward-restricted’ cluster exhibited reduced responsiveness to music (67%) and/or libido (67%) but heightened responsiveness to colour (92%) (all significantly different from the ‘reward-seeking’ and ‘control-like’ clusters, *P*_FDR_ < 0.05). The ‘eating-predominant’ cluster demonstrated significantly increased sweet tooth, appetite and responsiveness to music, and decreased libido and responsiveness to art compared with the control-like cluster (all *P*_FDR_ < 0.05); the prevalence of changes in non-primary reward behaviours in the ‘eating-predominant’ cluster also differed significantly from the ‘reward-seeking’ and ‘reward-restricted’ clusters (*P*_FDR_ < 0.05). The ‘control-like’ cluster showed minimally altered responsiveness in any of the sampled reward domains, similar to our healthy control group (see Table 1). These reward clusters mapped onto the two principal factors identified in the MCA (Fig. 1).

**Figure 4:**
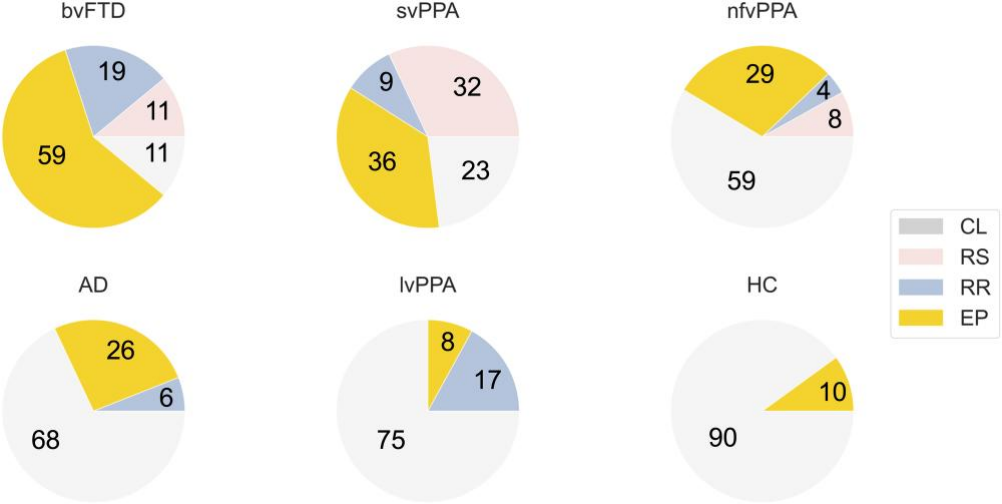
**Distribution of reward phenotypic clusters in dementia syndromes.** Pie charts show the percentage of cases exhibiting each reward behavioural phenotypic cluster (see Fig. 3) in each diagnostic group. The number of participants in each diagnostic group was as follows: 27 bvFTD, 34 AD, 12 lvPPA, 22 svPPA, 24 nfvPPA and 42 HC. AD, patient group with typical Alzheimer’s disease; bvFTD, patient group with behavioural variant frontotemporal dementia; CL, ‘control-like’ cluster; EP, ‘eating-predominant’ cluster; HC, healthy control group; lvPPA, patient group with logopenic variant primary progressive aphasia; nfvPPA, patient group with non-fluent/agrammatic variant primary progressive aphasia; RR, ‘reward-restricted’ cluster; RS, ‘reward-seeking’ cluster; svPPA, semantic variant primary progressive aphasia.

Reward behavioural phenotypes were variably represented across participant diagnostic groups (Fig. 4, Supplementary Table 6). Only a small proportion of healthy controls exhibited features of a non-‘control-like’ phenotype (‘eating-predominant’ in 10%). Abnormal reward behavioural phenotypes were represented in 89% of the bvFTD group and 77% of the svPPA group: while ‘eating-predominant’ was the commonest reward phenotype in both groups, around a third of svPPA patients had a ‘reward-seeking’ phenotype (more prevalent than in any other group), whereas the ‘reward-restricted’ phenotype was more prevalent in bvFTD than other groups. A majority of patients with AD, lvPPA and nfvPPA had a ‘control-like’ reward behavioural phenotype; among these syndromic groups, lvPPA was distinguished by a higher prevalence of the ‘reward-restricted’ phenotype, whereas ‘reward-seeking’ was not represented in the AD or lvPPA groups.

Further, reward behavioural clusters varied in the strength of their association with more general socio-emotional behavioural abnormalities (Fig. 3, Supplementary Table 6). Compared with the ‘control-like’ cluster, the ‘reward-seeking’ cluster had significantly increased prevalence of all sampled socio-emotional behaviours except apathy, the ‘reward-restricted’ cluster had significantly more frequent disinhibition and obsessionality and the ‘eating-predominant’ cluster had significantly more frequent disinhibition, obsessionality and loss of empathy (all *P*_FDR_ < 0.05).

Reward behavioural clusters did not differ significantly in the associated group demographics (age, gender, handedness, education) or disease severity indices (MMSE, illness duration) of their constituent participants (Supplementary Table 6).

### Neuroanatomical associations

**Figure 5:**
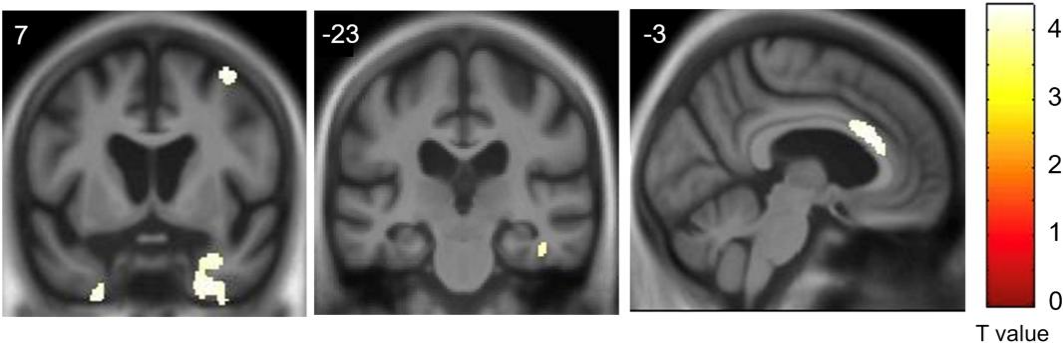
**Neuroanatomical substrate of abnormal reward behaviour.** Grey matter associations of the leading reward factor (factor 1) in the combined patient cohort were derived from a VBM analysis of patients’ brain MRI images (*n* = 96 scans; see text and Table 2 for details). Grey matter clusters were significantly associated with the factor at *P* < 0.05 after family-wise error correction over the whole brain. Statistical parametric maps have been rendered on coronal (left and middle) and sagittal (right) sections of the group mean template T_1_-weighted MRI brain image. Coordinates in Montreal Neurological Institute space are given for each section. A bar on the right shows the corresponding voxel-wise *T*-values. The right hemisphere is displayed on the right in the coronal sections.

Neuroanatomical associations identified in the VBM analysis are presented in Table 2 and Fig. 5.

**Table 2:** Neuroanatomical associations of reward behaviour in the combined patient cohort

Region	Side	Cluster (voxels)	Peak (mm)			*T* score	*P*_FWE_
			*X*	*Y*	*Z*		
Anterior cingulate gyrus	L	407	−6	26	22	4.34	0.018
Temporal pole	R	1198	30	14	−30	4.11	0.020
			26	4	−45	4.09	0.021
			32	−4	−40	4.00	0.029
	L	146	−27	4	−51	4.04	0.025
			−21	12	−44	3.94	0.036
Middle frontal gyrus	R	94	38	6	63	4.07	0.023
Fusiform gyrus	R	52	44	−22	−24	3.89	0.042

The table shows grey matter regions from the VBM analysis significantly positively associated with the leading reward factor (factor 1) over the combined patient cohort. Local maxima shown attained significance threshold (*P* < 0.05) after family-wise error correction for multiple voxel-wise comparisons over the whole brain. Coordinates are in standard Montreal Neurological Institute space. All clusters with extent larger than 50 voxels are presented.

At threshold *P*_FWE_ < 0.05 corrected for multiple comparisons over the whole brain, the first principal (negatively signed) reward factor over the combined patient cohort was significantly associated with regional grey matter (i.e. an increased prevalence of abnormal reward behaviour was associated with grey matter atrophy) in a distributed, anterior bi-hemispheric network. This network encompassed the anterior cingulate gyrus, both temporal poles, the right fusiform gyrus and the right middle frontal gyrus. No significant grey matter associations of the second principal reward factor were identified at the prescribed significance threshold.

## Discussion

Here we have shown that the diverse reward-oriented behavioural changes in FTD and AD syndromes are underpinned by two principal driving factors: these we characterize as a ‘gating’ factor that determines the emergence of altered reward behaviour, and an orthogonal ‘modulatory’ factor that determines how that behaviour is directed. These factors are expressed jointly in a set of core, multimodal hedonic phenotypes, variably represented across the FTD and AD spectrum and linked to more pervasive socio-emotional behavioural abnormalities. Emergence of aberrant reward behaviour in these diseases is associated with a common profile of grey matter atrophy, involving the cortical circuitry that mediates behavioural salience and semantic and affective appraisal of sensory stimuli. We now consider the implications that follow from these findings.

In line with previous evidence,^2,5,12,14,15,18,32,35,54^ alterations in reward behaviour were most frequent overall in bvFTD and svPPA and were exhibited by most patients in these syndromic groups. Nevertheless, changes in reward behaviour were also present in around two-thirds of patients in other syndromic groups, well in excess of healthy older controls. Behavioural changes directed toward primary rewards, particularly food, were leading hedonic features across the patient cohort, further corroborating previous work.^2,10,11^ However, behavioural changes directed toward non-primary rewards also occurred in most patients with bvFTD and svPPA, significantly more frequently than in other patient groups.

The resolution of the complexity of hedonic behavioural alterations to two principal driving factors aligns with current models of reward system physiology.^1,40,55^ Reward-directed behaviour in humans as in other animals depends fundamentally on an instigating neural state that is goal-oriented—this manifest potentially as shifts in physiological arousal, attention, anticipation and mental imagery as well as motor routines and a disposition to new learning.^40^ The corollary of this state is the ‘gating’ of neural resources into reward behaviour, irrespective of its precise object or direction: a ‘goad without a [specific] goal’. A change in this permissive state would correspond to a change in reward behaviour along factor 1 in our analysis. In general, however, reward behaviour is tuned to prioritizing particular goals while minimizing adverse outcomes—most fundamentally, behaviour may be directed toward appetitive approach or active avoidance.^1,40^ This ‘modulatory’ driver of reward behaviour operates orthogonally to the ‘gating’ driver and corresponds to factor 2 here. The interplay of these two factors gives scope for the development of different reward phenotypes, according to whether behavioural changes are more pervasive (‘reward-seeking’, ‘reward-restricted’ phenotypes) or less pervasive (‘eating-predominant’, ‘control-like’ phenotypes), and how those changes are directed (chiefly, the ‘reward-seeking’ versus ‘reward-restricted’ phenotypes).

The degree to which behaviour directed to particular reward objects can be modulated is likely to depend in part on how contingent those objects are on semantic, emotional and socio-cultural context. Food intake, for example, while clearly influenced by socio-cultural norms, is essential for homoeostasis; changes in food-seeking are, therefore, a potent index of a general shift in goal-oriented behaviour (factor 1 here) but less likely to drive the modulation of reward behaviour independently of other reward objects (factor 2 here). On the other hand, hedonic valuation of stimuli lacking intrinsic biological value (such as music, religion or art) depends heavily on active decoding and integration of often-ambiguous semantic and affective signals: processes intimately related to the interpretation of signals originating with other people.^56–58^ In this connection, sexual behaviour is an interesting instance of a powerful biological imperative that is also strongly contingent on interpersonal signal processing and socio-cultural norms.^1,15,16^ Disembodied colour, on the other hand, is the most abstract hedonic object surveyed here, and the least obviously possessing intrinsic biological value, social or other semantic associations;^59^ interestingly, responsiveness to colour and art were dissociated (Supplementary Table 7). Heightened colour responsiveness in the ‘reward-restricted’ cluster suggests that this phenotype reflects an abnormal redirection of hedonic energy away from more typical, biologically and/or socio-emotionally valuable reward objects, in patients with damaged semantic and affective decoding mechanisms.

The principal factor ‘gating’ altered reward behaviour here was correlated with atrophy of brain circuitry previously implicated in processing the salience of sensory stimuli and evaluating their semantic and affective significance, both in the healthy brain and in neurodegenerative disease.^1,6,31,60–63^ The present neuroanatomical findings were obtained after taking into account the atrophy profiles attributable to particular syndromic groups, but accord with previous evidence concerning the cerebral correlates of abnormal hedonic behaviour directed to food, sex, music and phobic objects in both FTD and AD.^9–11,15,16,18,25^ Anterior cingulate gyrus and temporal poles are hub zones, respectively, of the cerebral salience and semantic appraisal networks.^43,45,46^ The anterior cingulate is particularly involved in anticipation of reward and monitoring of reward prediction error,^60^ whereas the temporal poles integrate information about verbal and non-verbal (including socio-emotional) objects and concepts, including hedonic valence categories.^31,61,63–66^ Previous studies of svPPA and other neurodegenerative syndromes have emphasized the critical role of semantic impairments in abnormal reward processing.^6,11,12,18,32,36,67–69^ Further, a critical neuroanatomical substrate of anhedonia in svPPA encompasses the right temporal pole and anterior cingulate.^70^

Other, connected regions identified here amplify the functions of the cortical salience and semantic hubs. Non-dominant prefrontal cortex is integrally involved in initiating, monitoring and modulating behaviours related to self-schema and self-projection,^46,71–75^ plausibly accounting for its documented association with altered sexual behaviour in FTD.^15^ Fusiform gyrus is critical for dynamic visual object representations and the conjunction of semantic and affective features.^75,76^ A coherent, context-appropriate and adaptive behavioural response to hedonic stimuli is likely to rely on dynamic communication between all these regions and large-scale networks, particularly where the identity or value of the reward is implicit or ambiguous.^46,75^ If the generation of appropriate reward-oriented behaviour is envisaged as the output of a neural ‘template matching’ algorithm that links incoming sensory data with stored hedonic representations,^33^ then inappropriate activation of the representation (impaired salience coding) or a degraded template (impaired semantic appraisal) could equally lead to inappropriate reward valuation and a maladaptive behavioural response. Aberrant representations of own interoceptive, somatosensory or emotional states would feed into this process.^46,77–79^

Given the neuroanatomical correlates here, the more severe and convergent hedonic phenotypes of bvFTD and svPPA follow predictably from the known canonical (and overlapping) network pathologies of these syndromes.^21,43,45,80,81^ However, behavioural abnormalities may be generated by dysfunctional interactions between coupled brain networks as well as targeting of networks *per se.* Thus, attenuated salience network activation would tend to promote anticorrelated over-activation of the coupled default mode network (a putative mechanism for the emergence of artistic proclivities in FTD),^23^ whereas conversely, in AD relatively intensified detection of salient negative environmental and socio-emotional signals might lead to heightened sensitivity to punishment.^4,14,18^ Abnormal functional connectivity that is not reflected in a discrete atrophy profile may also account for the lack of a VBM signal here in striatum or insula, which on face value is surprising, given previous evidence implicating these structures in aberrant reward processing in FTD and AD.^10,11,41,49^ It may also be relevant that our study sampled diverse non-primary as well as primary rewards: it is not clear to what extent these share subcortical neural substrates.

Associations between altered reward responsiveness and other abnormal socio-emotional behaviours should be interpreted cautiously. Disinhibition and obsessionality, here associated with more prominently abnormal reward behaviour, might plausibly give rise to altered reward responsiveness as part of a broader repertoire of behavioural dysregulation with impaired detection and integration of salient socio-emotional and interoceptive signals.^5,27,28^ However, these are themselves complex and multidimensional phenomena, and how they are linked to abnormal reward processing has not been established. Inappropriate goal-setting or impaired satiety processing resulting from aberrant hedonic valuation could directly promote or perpetuate obsessive and/or disinhibited behaviours, while erosion of the conceptual lexicon might restrict the repertoire of potentially rewarding objects to a few highly familiar items that are then pursued obsessively and exclusively (exemplified by food faddism and musicophilia accompanying svPPA or monochromatic fashion sense in some patients with bvFTD).^8,11,17,19,35^ Reduced awareness of one’s own or deficient inferences about others’ hedonic goals could impair empathy and humour (indeed, much humour inheres in the implicit recognition of thwarted intent or desire).^30,82^ It is also noteworthy that apathy was commonly observed across reward phenotypes (Supplementary Table 6): however, while anhedonia and apathy are significant and potentially dissociable issues in FTD syndromes,^47–49^ we did not observe a uniformly ‘anhedonic’ apathetic phenotype. This may, at least in part, reflect our survey methodology: for example, some patients included in the ‘control-like’ cluster may have had a general indifference to reward that was not reflected in any strong behavioural aversion.

From a clinical perspective, our findings highlight that altered behaviour directed toward non-primary as well as primary rewards define the striking hedonic phenotypes of bvFTD and svPPA; indeed, in both syndromes, the second most commonly affected hedonic domain was music (chiefly manifesting as musicophilia). In addition, relatively group-specific behavioural signatures were identified (diminished libido and altered responsiveness to art and colours in bvFTD; increased religiosity in svPPA). Together, the findings indicate that certain changes in reward behaviour—in particular, marked reward-seeking behaviours and changes directed toward non-primary rewards—are more likely to constitute markers of FTD than AD. However, there was substantial variability in the hedonic phenotype exhibited by individual patients with particular dementia syndromes. Hedonic phenotypes were trans-diagnostic, in the sense that no single reward behavioural cluster was exclusive to a diagnostic syndrome, while no syndrome conformed to a single cluster (Fig. 4, Supplementary Table 6). Although some phenotypic features overlapped between reward clusters (e.g. sweet tooth and altered appetite), clusters did not differ in illness duration, severity or other general clinical or demographic factors (Supplementary Table 6), suggesting they constitute distinct profiles of reward circuit dysfunction, rather than simply stages on a continuum. A small proportion of our healthy older control group also showed changes in reward behaviour: while information remains limited, this is consistent with previous evidence for altered appetite and dietary preferences in healthy ageing.^35,83^ Within the FTD spectrum, reward-seeking behaviours were a particular hallmark of svPPA, while reduced reward responsiveness or a focus on unusual objects (such as colours) pointed to bvFTD, in line with recent formulations.^12^

Our findings and the synthesis we propose should be regarded as provisional, pending further substantiation. The limitations of this study suggest directions for future work. The reward behaviours sampled here are signposts in a much richer hedonic landscape. Future studies should address a wider variety of reward objects (in particular, money, interpersonal affinity and prosociality), to establish how well these can be fitted within the framework proposed here. This is particularly important for behaviour directed toward non-primary rewards that might be contingent on availability and/or prevailing cultural values. More granularity is required to capture and quantify the spectrum of changes within hedonic domains (appetite alterations, for example, may include food faddism as well as hyperphagia or anorexia; reduced responsiveness to music or art ranges from indifference to active avoidance). Altered reward behaviours could be quantified on metrics such as frequency and intensity, and patients’ self-reports compared with those of informants, to assess changes in awareness of own hedonic goals and behaviour. Caregiver burden could be assessed as a potentially important modulatory factor. Our study rests on an experimenter-imposed survey of a single, possibly unrepresentative cohort: the generalizability of our findings remains to be established. This will await the development of a validated, multidimensional instrument to quantify the severity of behavioural changes in a wider range of hedonic domains—as well as multi-centre collaboration, to apply that instrument systematically across more socio-culturally diverse populations. Future studies should additionally examine the evolution of hedonic phenotypes longitudinally: this would help to define how the proposed reward behavioural clusters relate to one another, as well as to other socio-emotional behaviours and disease characteristics.

Behavioural measures should be supplemented with neuro-economic cognitive paradigms, autonomic recordings and neuro-hormonal assays to establish both the subjective correlates and physiological mechanisms of reward behaviours and their component subprocesses. Models for such paradigms and for experimental verification of reported changes in reward behaviour are available.^11,29^ Behavioural phenomena such as disinhibition and apathy could be assessed using standardized instruments such as the Cambridge Behavioural Inventory^84^ but themselves require experimental deconstruction to determine which facets are most relevant to altered reward behaviour and how these may link together mechanistically. This in turn will demand more comprehensive pathophysiological models of complex behavioural change in neurodegenerative disease. A full delineation of the underlying neural mechanisms will require functional neuroimaging paradigms exploiting techniques (such as functional magnetic resonance imaging and magnetoencephalography) that can capture dynamic, integrative processes. Indeed, the lack of a structural neuroanatomical correlate of our second principal, ‘modulatory’ driver may indicate that this factor is expressed in a shifting pattern of functional connectivity within the distributed network identified here.^66^ It remains unclear whether hedonic behaviour is entirely determined by brain network topography, or whether molecular pathology imprints the phenotype: studying genetically mediated syndromes will be especially pertinent to this question. Combining functional neuroimaging with pharmaco-modulatory paradigms promises to elucidate the role of dopamine, gamma-aminobutyric acid and other neurotransmitters in the genesis of adaptive and maladaptive reward-oriented behaviour from neural circuitry in neurodegenerative diseases,^85,86^ which may in turn inform novel symptomatic therapies.^87^ Ultimately, neuropathological correlation will be required, particularly to dissect the nosological diversity of FTD, which could potentially be expressed as distinct, molecularly determined hedonic phenotypes.

Changes in reward behaviour—particularly those affecting non-primary rewards—are not typically addressed in standard neurological history taking or neuropsychological assessments. Beyond their neurobiological interest, the present findings call for a more searching bedside appraisal of these existential manifestations of dementia, in the service of earlier diagnosis, more engaging management and a fuller understanding of the impact of these diseases on patients’ daily lives.

## Supplementary Material

fcad027_Supplementary_Data

## Data Availability

The data that support the findings of this study are available on reasonable request from the corresponding author. The data are not publicly available in line with the terms of the original ethics approval.

## Abbreviations

AD =: Alzheimer’s disease
bvFTD =: behavioural variant frontotemporal dementia
FTD =: frontotemporal dementia
lvPPA =: logopenic variant primary progressive aphasia
MCA =: multiple correspondence analysis
MMSE =: Mini-Mental State Examination
nfvPPA =: non-fluent variant primary progressive aphasia
*P*_FDR_ =: false discovery rate corrected *P*-value
*P*_FWE_ =: family-wise error corrected *P*-value
PPA =: primary progressive aphasia
svPPA =: semantic variant primary progressive aphasia
VBM =: voxel-based morphometry
