# Supplementary material for: The architecture of abnormal reward behaviour in dementia: multimodal hedonic phenotypes and brain substrate
Source: Brain Commun. 2023 Feb 9;5(2):fcad027. doi: 10.1093/braincomms/fcad027 (PMC10023829; doi:10.1093/braincomms/fcad027)
Supplement: fcad027_Supplementary_Data [file fcad027_supplementary_data.pdf]

SUPPLEMENTARY MATERIAL

The architecture of abnormal reward processing in dementia: multimodal hedonic phenotypes and brain substrate,

by A Chokesuwattanaskul et al

Multiple correspondence analysis: background and rationale

Multiple correspondence analysis (MCA) is a type of exploratory factor analysis specifically designed for categorical datasets, analogous to principal component analysis on continuous datasets. One distinct advantage of MCA is that it does not assume any particular distribution of the data. MCA projects the data onto a lower dimensional space that maximally retains the information of the initial data, and allows simultaneous examination of all potentially relevant variables (a further advantage over conventional pair-wise associations). Because MCA is conducted at the level of response categories rather than variables, it allows the relationship between each response category and variable to be characterised. For example, if there are three types of responses (increased, decreased, no change) for a particular reward behaviour, MCA will generate three variables to represent these response categories, represented in an 'indicator matrix', where each row corresponds to a participant and each column represents a response category.

After the indicator matrix is derived, it is transformed into a probability matrix where each element is divided by the grand sum of the matrix, hence the name. The final step is to apply singular vector decomposition on the "standardised" probability matrix, to generate the eigenvectors of rows and columns and a matrix of eigenvalues. Intuitively, eigenvectors of this standardised probability represent the explained variance (information) in different, orthogonal dimensions. We reduce the dimension to match the desired number of factors: the eigenvectors and eigenvalues, standardised by the magnitude of the sum of the row and column values in the probability matrix, are used to generate row and column factor scores.

Each factor acts as a 'latent variable' that explains a portion of the variance in the data. The first factor accounts for the largest portion of explained variance; each subsequent factor is orthogonal to the former factors and describes a portion of the remaining variance. In general, the 'elbow' on the scree plot of explained variance is used to determine the number of factors to retain. Additionally, it is recommended that all retained factors together explain > 70% of the total variance ('inertia'). Here, the principal factors 1 and 2 are column factors: they represent a portion of variance in the column dimension of the data. The correlation (squared cosine) of a feature on a particular factor quantifies the strength of association

between them - a measure of the quality of representation of the feature by that factor, or how well the feature (presence vs. absence) is discriminated by the factor. Higher squared cosine values correspond to greater discriminatory power. The sum of squared cosine values across all retained factors denotes how well each feature is represented by the retained factors (normalised between 0 and 1, a value closer to 1 signifying that the feature is well represented).

Another useful property of MCA is that it allows additional data not included explicitly in the original analysis to be mapped as "supplementary data" onto the same dimensions as the original model. In other words, MCA allows us to map new data points, whether a new observation or a new feature (here, diagnostic groups), into the common, derived factor space. We can derive the factor values for the supplementary feature by exploiting the information derived from singular vector decomposition. If the original features are represented in the matrix f(COL) x f(ROW), a supplementary feature, c_1, is mapped into the column factor space as: f(COL+ c_1) x f(ROW); the additional information will be f(c_1) x f(ROW)). Such supplementary features do not affect the factor analysis of the original data, however, this process allows them to be assessed (and visualised) in a common space with the original features.

Cluster stability analysis: background

Cluster stability analysis[1] is a form of sensitivity analysis used for evaluating the performance of clustering algorithms. Here, we employed a bootstrapping technique that subsamples a designated proportion (here, 80%) of data from the whole dataset, with replacement after each iteration. The k-means clustering algorithm was applied on all subsampled data. In each iteration, the similarity of the clustering result on the subsample with the entire original dataset was determined by calculating the mean percentage of participants in each cluster who belonged to the same cluster in the original analysis, across all clusters (see Supplementary Figure 3). A similarity of 100% would signify all participants in each cluster in the subsample were included in the same clusters in the original analysis on the entire dataset. A cluster stability index was derived by averaging the percentage similarity of every subsample over the assigned number of iterations.

Brain imaging acquisition and pre-processing

For each patient, a sagittal 3D magnetization-prepared rapid-gradient echo T1-weighted volumetric brain MRI sequence (TE/TR/TI 2.9/2200/900ms, dimensions 256 × 256 × 208, voxel volume of 1.1 × 1.1 × 1.1mm) was acquired on a Siemens Prisma 3T MRI scanner using a 32- channel phased-array head-coil and pre-processed using standard procedures in SPM12 (www.fil.ion.ucl.ac.uk/spm, details in

Supplementary material). Ninety-six volumetric brain MRI scans from the patient cohort (22 bvFTD, 23 AD, 20 nfvPPA, 20 svPPA, 11 lvPPA) were included in the VBM analysis. Twenty-three patients were excluded either because their scan was unavailable or of inadequate quality. Pre-processing of brain images was performed using the New Segment and Diffeomorphic Anatomical Registration Through Exponentiated Lie Algebra (DARTEL) toolboxes on SPM12, following an optimised protocol. Normalisation, segmentation, and modulation of grey and white matter images were performed using default parameter settings. Grey matter images were smoothed using a 6 mm full-width-at-half-maximum Gaussian kernel. A study-specific template brain image was created by warping all bias-corrected native space brain images to the final DARTEL template and calculating the average of the warped brain images. Total intracranial volume for each participant was calculated by summing grey matter, white matter and cerebrospinal fluid volumes.

Supplementary Table 1. Neuropsychological and general behavioural characteristics of participant groups

Cognitive domain	Controls	AD	lvPPA	bvFTD	svPPA	nfvPPA
	n = 42	n = 34	n = 12	n = 27	n = 22	n = 24
**General**						
WASI VIQ	125.5 (120.2-129.8)[d]	**97.0 (84.0-110.5)[2,4,e]**	**55.0 (55.0-70.0)[1,a]**	**76.0 (55.0-113.0)[b]**	**65.0 (55.0-78.8)[1,b]**	**74.0 (66.0-9.05)[c]**
WASI PIQ	121.0 (113.0-128.8)[c]	**83.0 (74.0-96.2)[4,e]**	**85.0 (74.0-94.5)[4,a]**	**94.0 (77.0-109.0)[4,b]**	116.0 (96.8-129.5)[1,2,3,5,b]	**89.0 (77.0-106.0)[4,c]**
**Episodic memory**						
RMT Faces (/50)	44.0 (41.0-48.0)[b]	**30.5 (26.0-35.2)[m]**	**29.0 (25.0-32.5)[a]**	**30.5 (25.0-37.0)[a]**	**30.0 (28.0-36.5)[d]**	**34.0 (30.0-39.0)[c]**
RMT Words (/50)	49.0 (47.8-50.0)[b]	**28.5 (25.0-38.2)[m]**	**25.0 (25.0-38.0)[a]**	**36.0 (28.8-44.2)[c]**	**33.0 (28.5-39.0)[f]**	**41.0 (28.0-46.0)[c]**
**Executive**						
DS forward (max 12)	7.0 (6.0-8.0)[b]	6.0 (5.0-7.0) [2,5,e]	**3.0 (1.0-4.0)[1,3,4,a]**	6.0 (5.0-7.8)[2,5,a]	**7.0 (6.0-7.2)[2,5,b]**	**4.0 (4.0-5.0)[1,3,4,c]**
DS reverse (max 12)	5.0 (4.0-6.0)[b]	**4.0 (3.0-4.5)[2,4,5,f]**	**2.0 (0-3.0)[1,3,4,a]**	**4.0 (3.0-5.0)[2,5,a]**	5.0 (4.0-5.0)[1,2,5,b]	**3.0 (0-3.0)[1,3,4,c]**
DKEFS Stroop: colour (90 sec)	30.0 (27.0-35.0)[c]	**54.0 (45.0-61.0)[2,5,h]**	90.0 (78.5-90.0)[1,3,4,a]	**49.0 (36.0-74.0)[2,5,b]**	**53.0 (39.0-71.5)[2,5,b]**	**90.0 (63.0-90.0)[1,3,4,g]**
words (90 sec)	22.0 (20.0-25.0)[c]	**32.0 (28.0-36.0)[5,h]**	**47.0 (41.0-55.0)[3,4,a]**	**30.0 (23.0-36.0)[2,5,b]**	28.0 (23.0-38.2)[2,5,b]	**66.0 (52.0-90.0)[1,3,4,g]**
interference (180 sec)	54.0 (45.5-67.0)[c]	**148.0 (106.0-180.0)[h]**	**180.0 (180.0-180.0)[3,4,a]**	**92.0 (57.0-180.0)[2,b]**	**92.5 (61.2-131.2)[2,5,b]**	**180.0 (119.0-180.0)[4,g]**
TMT-A (sec)	29.5 (23.8-36.2)[b]	**69.0 (58.0-127.0)[4,f]**	**106.0 (58.5-141.5)[4,a]**	**56.0 (40.0-149.0)[b]**	**48.5 (33.0-61.8)[1,2,b]**	**62.0 (44.0-143.0)[g]**
TMT-B (sec)	58.0 (47.0-83.2)[b]	**300.0 (194.5-300.0)[4,f]**	**300.0 (300.0-300.0)[4,b]**	**200.0 (100.0-300.0)[b]**	**105.0 (82.8-156.5)[1,2,b]**	**238.0 (150.0-300.0)[g]**
Letter fluency (F)	18.0 (15.0-20.2)[b]	**10.0 (6.0-13.0)[2,f]**	**2.0 (0-4.5)[1,a]**	**6.0 (2.0-12.0)[b]**	**6.5 (0.8-12.2)[b]**	**3.0 (0-8.2)[h]**
Category fluency (animals)	24.0 (21.0-28.0)[b]	**9.0 (5.8-13.2)[e]**	**3.0 (1.0-6.5)[a]**	**9.0 (4.0-17.0)[b]**	**4.5 (1.8-9.5)[b]**	**8.5 (1.5-14.5)[h]**
**Language**						
BPVS (/150)	148.0 (148.0-149.0)[b]	**144.0 (124.8-146.2)[4,e]**	**132.0 (79.5-145.0)[a]**	**140.0 (101.0-148.0)[b]**	**56.5(19.5-105.5)[1,3,5,b]**	**139.5 (114.8-144.0)[4,b]**
GNT (/30)	26.0 (23.8-28.0)[b]	**14 (4.5-20.5)[4,f]**	**7.5 (0-17.8)[4,b]**	**14.0 (2.2-23.8)[a]**	**0 (0-0)[1,3,5,b]**	**9.0 (5.0-19.0)[4,c]**
**Other skills**						
GDA (/24)	14.0 (11.8-19.0)[b]	**2.0 (1.0-6.5)[4,f]**	**0 (0-0.5)[3,4,a]**	**6.0 (3.5-13.0)[2,d]**	12.5 (3.5-17.0)[1,2,5,b]	**3.0 (0-5.0)[4,c]**
VOSP (/20)	19.0 (18.0-20.0)[b]	**16.0 (14.0-17.2)[e]**	**15.0 (14.0-16.5)[a]**	**15.5 (10.0-18.0)[c]**	**16.0 (15.0-17.5)[c]**	**16.5 (14.0-17.2)[d]**
**General behavioural change**						
Disinhibition	1 (2)	**8 (24)[3,4]**	2 (17)[3,4]	**24 (89)[1,2,5]**	**15 (68)[1,2,5]**	**7 (29)[3,4]**
Apathy	2 (5)	**23 (68)**	**5 (42)[3]**	**23 (85)[2,4,5]**	**10 (45)[3]**	**12 (50)[3]**
Obsessionality	1 (2)	**8 (24)[3,4]**	3 (25)[3]	**21 (78)[1,2,5]**	**14 (64)[1]**	**8 (33)[3]**
Loss of empathy	0	**6 (18)[3,4,5,]**	**6 (50)**	**23 (85)[1]**	**14 (64)[1]**	**14 (58)[1]**
Inappropriate humour	4 (10)	3 (9)[2,3,4,5]	**5 (42)[1,4]**	**21 (78)[1]**	**19 (86)[1,2]**	**17 (71)[1]**

Counts (percentage of group) are shown for behavioural change data; and mean (standard deviation) or median (interquartile range) scores are shown for neuropsychological tests (with maximum scores in parentheses). Differences between diagnostic groups were assessed using ANOVA, Kruskal-Wallis test and chi-square test with post-hoc correction. Significant differences between patient groups and healthy controls are in bold; significant differences between patient groups are coded as follows: [1]significantly different from AD, [2]significantly different from lvPPA, [3]significantly different from bvFTD, [4]significantly different from svPPA, [5]significantly different from nfvPPA (all $p_{FDR} < 0.05$). AD, patient group with typical Alzheimer's disease; BPVS, British Picture Vocabulary Scale (Dunn, Dunn and Whetton, 1982); bvFTD, patient group with behavioural variant frontotemporal dementia; Controls, healthy control group ; D-KEFS, Delis Kaplan Executive System (Delis et al., 2001); DS, Digit Span; GDA, Graded Difficulty Arithmetic test (Jackson and Warrington, 1986); GNT, Graded Naming Test (McKenna and Warrington, 1983); lvPPA, patient group with

logopenic variant primary progressive aphasia; nfvPPA, patient group with nonfluent/agrammatic variant primary progressive aphasia; PIQ, performance IQ; RMT, Recognition Memory Test (Warrington, 1984); svPPA, patient group with semantic variant primary progressive aphasia; TMT, trail making test; VIQ, verbal IQ; VOSP, Visual Object and Space Perception Battery – Object Decision test (Warrington, McKenna and Orpwood, 1998); WASI, Wechsler Abbreviated Scale of Intelligence (Wechsler, 1997). A reduced number of participants completed certain tests, as follows: [a]n-1, [b]n-2, [c]n-3, [d]n-4, [e]n-5, [f]n-6, [g]n-7, [h]n-8, [m]n-13.

Supplementary Table 2. Symptom survey used to record changes in reward behaviour

Reward domain	Questions	Coding
	For each question, please say whether [your / his / her] liking, enjoyment and/or interest has changed, now compared with 10 years ago	
Primary		
Appetite	Has there been a change in appetite/ portion size consumed at meals?<br>If yes, is this increased or decreased?<br>Comment:	Yes / No<br>Inc / Dec
Sweet tooth	Has there been any increased liking for sweet foods?<br>Comment:	Yes / No
Sexual behaviour	Has there been any change in sexual interest or activity?<br>If yes, is this increased or decreased?<br>Comment:	Yes / No<br>Inc / Dec
Non-primary		
Music	Has there been any change in interest or feelings in response to music?<br>If yes, is this increased or decreased?<br>Comment:	Yes / No<br>Inc / Dec
Religion	Has there been any increased religious interest or feelings?<br>Comment:	Yes / No
Art	Has there been any change in interest in viewing or producing visual art?<br>If yes, is this increased or decreased?<br>Comment:	Yes / No<br>Inc / Dec
Colours	Has there been any increased preference or appreciation for particular colours?<br>Comment:	Yes / No

Respondents were patients' primary caregivers or healthy control participants. Prior to completing the survey, caregivers were instructed that a relevant behavioural 'change' in a particular reward domain might comprise an evident alteration in liking, enjoyment and/or interest (e.g., seeking or avoidance of the relevant item) that the caregiver had observed in the person with dementia.

Supplementary Table 3. Caregiver comments extracted from the reward behavioural symptom survey

Reported behavioural change	Syndrome	Representative comments
Primary rewards		
Altered appetite	bvFTD	Always wants to eat toast and peanut butter
	svPPA	Always wants to eat tomato ketchup
Increased liking for sweet foods	AD	Likes chocolate, sweets, ice cream
	bvFTD	Always eats sweet foods, biscuits, cakes, grapes
	svPPA	Likes sweets, scones, tomato ketchup
	nfvPPA	Likes sweets, jams, tarts, chocolate
Increased sexual interest or activity	bvFTD	Increased libido, more sexual feelings, more interested in pornography and masturbation
	svPPA	Increased libido, more physical (cuddling), overly affectionate
Decreased sexual interest or activity	AD	Does not initiate sexual activity, less libido
	lvPPA	Decreased libido
	bvFTD	Decreased libido, no sexual behaviour at all, no interest at all
	svPPA	Decreased libido
Non-primary rewards		
Increased responsiveness to music	bvFTD	Listen to music more<br>Loves music and dancing<br>Really like certain songs
	svPPA	Likes older music more<br>Listens to music compulsively, gets very emotional<br>Likes music more<br>Likes listening to the same few things
	nfvPPA	Listens repetitively to same music
Decreased responsiveness to music	bvFTD	Now averse to music
	svPPA	Less interested in music
Increased religiosity	lvPPA	Starts going to church after stopped going 20 years previously
	bvFTD	Obsessed with saying grace before meals<br>Prays more regularly, more connected with God<br>Discusses religious topics more
	svPPA	Prays more, feels more 'in touch' with God<br>More committed to church<br>Now believes in afterlife<br>Speaks more about God<br>Now likes attending mass
	nfvPPA	Now watches religious TV programs
Increased interest in art	lvPPA	More into painting
	bvFTD	Recently got paid for work with a painting<br>Likes colouring book
Decreased interest in art	lvPPA	Studied history of art but less bothered now
	svPPA	No interest now though used to work at an art school

	nfvPPA	Doesn't like galleries anymore
Increased liking for particular colour(s)	AD	Likes green
	lvPPA	Prefer bright colours<br>Likes orange
	bvFTD	Loves bright colours<br>Likes pastel colours<br>Loves black and orange; loves green and orange
	svPPA	Always dresses in black<br>Dresses in one colour – red, purple, green<br>Loves red and black, hates green<br>Loves bright colours
	nfvPPA	Likes black more<br>Paints with more pastel colours

Representative comments from primary caregivers completing the symptom survey about patients' reward behaviours are presented here. AD, patients with typical Alzheimer's disease; bvFTD, patients with behavioural variant frontotemporal dementia; lvPPA, patients with logopenic aphasia; nfvPPA, patients with progressive non-fluent aphasia; svPPA, patients with semantic dementia.

Supplementary Table 4. Correlations of reward features with principal factors

	Factor score		Squared cosine		Sum of squared cosines
**Reward features**	**Factor 1**	**Factor 2**	**Factor 1**	**Factor 2**	
Appetite (Dec)	-0.26	-0.02	0.87	0.004	0.872
Appetite (Inc)	-0.37	0.00	0.91	0.000	0.909
Sweet tooth (Inc)	-0.31	-0.01	0.95	0.000	0.950
Sexual behaviour (Dec)	-0.54	-0.17	0.90	0.095	0.997
Sexual behaviour (Inc)	-0.66	0.42	0.69	0.281	0.972
Music (Dec)	-0.46	-0.31	0.67	0.296	0.965
Music (Inc)	-0.38	0.22	0.76	0.24	0.999
Art (Dec)	-0.53	-0.11	0.95	0.041	0.995
Art (Inc)	-0.37	-0.02	0.83	0.003	0.833
Colour (Inc)	-0.32	-0.23	0.64	0.326	0.970
Religion (Inc)	-0.47	0.29	0.70	0.269	0.971
**Clusters**					
‘Reward-seeking’	-0.37	0.180			
‘Reward-restricted’	-0.32	-0.180			
‘Eating-predominant’	-0.08	0.001			
‘Control-like’	0.20	0.004			

This table displays the squared cosine value for each reward feature with the two principal factors (factor 1 and factor 2) identified from the multiple correspondence analysis. The sum of the squared cosines from factor 1 and factor 2 for each feature is shown in the column ‘Sum’. Features with a high sum of squared cosine values (sum close to 1) are well-represented by the two principal factors. Dec, decreased; Inc, increased.

Supplementary Table 5. Associations of principal reward factors with general disease characteristics and socio-emotional behaviours

Characteristic	Factor 1 score			Factor 2 score		
General disease	**Spearman's rho**		**p-value**	**Spearman's rho**		**p-value**
Symptom duration	-0.16		0.08	-0.05		0.60
MMSE score	-0.04		0.69	0.11		0.23
General socio-emotional behaviours	**Present**	**Absent**		**Present**	**Absent**	
Disinhibition	-0.203 (-0.359 - (-)0.063)	0.080 (-0.058 - 0.202)	< 0.001	0.003 (-0.043 - 0.067)	0.004 (-0.003 - 0.004)	< 0.001
Apathy	-0.074 (-0.267 - 0.088)	0.077 -0.063 - 0.202)	< 0.001	0.001 (-0.044 - 0.020)	0.004 (0.001 - 0.004)	< 0.001
Obsessionality	-0.203 (-0.343 - (-)0.063)	0.080 (-0.053 - 0.202)	< 0.001	0.001 (-0.085 - 0.072)	0.004 (0.001 - 0.004)	< 0.001
Loss of empathy	-0.095 (-0.320 - 0.066)	0.080 (-0.063 - 0.202)	< 0.001	0.004 (-0.023 - 0.037)	0.001 (-0.003 - 0.004)	< 0.001
Inappropriate humour	-0.095 (-0.320 - 0.080)	0.080 (-0.063 - 0.202)	< 0.001	0.004 (-0.037 - 0.023)	0.001 (-0.003 - 0.004)	< 0.001

The table summarises the association of principal factors 1 and 2 with MMSE score and general socio-emotional behaviours across the combined patient cohort. Spearman's rho correlation coefficients and associated p values are shown for MMSE score; median (interquartile range) values are shown for patient subgroups reporting presence vs absence of each socio-emotional behaviour, together with p values of each subgroup comparison (assessed using Mann-Whitney U tests). MMSE, Mini-Mental State Examination score.

Supplementary Table 6. Demographic, clinical and neuropsychological characteristics of patients in each reward behavioural cluster

Characteristic	Reward-seeking	Reward-restricted	Eating-predominant	Control-like
	n = 12	n = 12	n = 41	n = 54
**General**				
No. (m:f)	7:5	8:4	29:12	31:23
Handed (R:L)	12:0	11:1	37:4	51:3
Age (y)	70.7 (8.1)	70.9 (8.1)	66.3 (7.1)	66.6 (7.7)
Education (y)	13.0 (10.8-16.0)	13.0 (12.0-16.5)	16.0 (12.0-16.0)	15.5 (12.0-16.0)
Illness (y)	5.6 (5.0-7.4)	5.7 (4.1-7.6)	5.0 (4.2-6.4)	4.7 (3.5-5.7)
**Syndromic diagnosis**				
AD	0 (0)	2 (17)	9 (22)	23 (43)
lvPPA	0 (0)	2 (17)	1 (24)	9 (17)
bvFTD	3 (25)	5 (42)	16 (39)	3 (6)
svPPA	7 (58)	2 (17)	8 (20)	5 (9)
nfvPPA	2 (17)	1 (8)	7 (17)	14 (26)
**Neuropsychological assessment**				
General				
MMSE (/30)	22.0 (20.2-26.0)	21.5 (15.0-26.5)	22.0 (16.0-28.0)	22.5 (14.2-27.0)
WASI VIQ	66.0 (55.0-74.0)[a]	88.0 (65.5-111.0)[a]	75.0 (55.0-105.0)[d]	83.5 (66.2-98.0)[d]
WASI PIQ	97.0 (91.5-108.0)[a]	94.0 (73.0-107.5)[a]	84.0 (73.0-110.0)[d]	89.5 (77.5-105.0)[d]
Episodic memory				
RMT Faces (/50)	28.0 (25.5-31.5)[a]	30.0 (25.0-33.0)[c]	30.0 (25.0-37.0)[d]	32.0 (27.0-36.2)[j]
RMT Words (/50)	30.0 (25.5-36.5)[b]	33.0 (25.0-44.0)[c]	33.0 (25.0-42.0)[h]	36.5 (27.8-44.0)[j]
Executive functions				
DS forward (max 12)	6.0 (6.0-6.8)[b]	5.0 (4.0-6.5)[a]	7.0 (5.0-7.5)[b]	5.0 (4.0-6.0)[d]
DS reverse (max 12)	5.0 (3.2-5.0)[b]	3.0 (3.0-4.5)[a]	4.0 (2.5-5.0)[b]	3.0 (2.0-4.0)[e]
DKEFS Stroop:<br>colour (90 sec)	45.5 (41.2-54.0)[b]	50.0 (39.5-76.0)[a]	59.0 (44.5-87.5)[b]	62.5 (49.0-85.0)[l]
words (90 sec)	30.5 (26.5-37.0)[b]	33.0 (26.5-71.0)[a]	36.0 (26.2-56.8)[c]	36.0 (29.0-55.0)[k]
interference (180 sec)	82.5 (59.0-96.8)[b]	145.0 (63.5-180.0)[b]	131.0 (77.5-180.0)[b]	180.0 (106.5-180.0)[k]
TMT-A	57.0 (42.5-73.5)[a]	53.0 (40.5-137.0)[a]	61.5 (41.8-144.5)[e]	65.0 (49.2-122.2)[h]
TMT-B	177.0 (97.5-300.0)[a]	180.0 (95.0-300.0)[a]	291.0 (108.8-300.0)[e]	290.0 (143.0-300.0)[i]
Letter fluency (F)	6.0 (3.2-8.5)[b]	9.0 (2.8-12.0)[b]	6.0 (1.0-12.5)[b]	6.0 (2.8-10.0)[j]
Category fluency (animals)	5.0 (2.2-8.2)[b]	8.0 (4.2-15.2)[b]	6.0 (2.0-12.0)[b]	9.0 (3.0-13.0)[i]
Language				
BPVS (/150)	**66.0 (38.5-106.0)[2,3,a]**	144.0 (121.5-148.0)[a]	135.5 (84.0-144.5)[1,c]	140.0 (120.2-146.0)[d]
GNT (/30)	**0 (0-1.5) [2,3,a]**	18.0 (4.0-24.0) [a]	10.0 (2.0-20.0) [1,d]	8.0 (2.0-19.0)[e]
Other skills				
GDA (/24)	5.5 (3.3-13.2)[b]	4.0 (0.5-7.5)[a]	4.0 (1.0-15.0)[d]	3.0 (0-8.0)[f]
VOSP (/20)	15.0 (13.5-15.5)[a]	16.0 (14.0-18.0)[a]	16.0 (13.0-18.0)[c]	16.0 (14.0-18.0)[h]
**General behavioural change**				
Disinhibition	**11 (92)**	**8 (67)**	**27 (66)**	10 (19)
Apathy	7 (58)	9 (75)	31 (76)	26 (48)
Obsessionality	**10 (83)**	**11 (92)**	**27 (66)**	6 (11)
Loss of empathy	**10 (83)**	8 (67)	**27 (66)**	18 (33)
Inappropriate humour	**11 (92)**	7 (58)	24 (59)	23 (43)

Counts (standard deviation) are shown for general demographic and clinical data; counts (percentage of group) are shown for diagnostic syndromes and behavioural change data; and mean (standard deviation) or median (interquartile range) scores are shown for neuropsychological tests (with maximum scores in parentheses). Differences between reward behavioural clusters were assessed using ANOVA, Kruskal-Wallis test and chi-square test with post-hoc correction. Significant differences ($p_{FDR} < 0.05$) compared with the 'control-like' cluster are in bold; significant differences compared with other reward clusters are coded as follows: [1]significantly different from 'reward-seeking' cluster, [2]significantly different from 'reward-restricted' cluster, [3]significantly different from 'eating-predominant' cluster (all $p_{FDR}<0.05$). AD, patient group with typical Alzheimer's disease; BPVS, British Picture Vocabulary Scale (Dunn, Dunn and Whetton, 1982); bvFTD, patient group with behavioural variant frontotemporal dementia; Controls, healthy control group; D-KEFS, Delis Kaplan Executive System (Delis et al., 2001); DS, Digit Span; f, female; GDA, Graded Difficulty Arithmetic test (Jackson and Warrington, 1986); GNT, Graded Naming Test (McKenna and Warrington, 1983); Handed, handedness; Illness, estimated symptom duration; L, left; lvPPA, patient group with logopenic variant primary progressive aphasia; m, male; MMSE, Mini-Mental State Examination score (Folstein, Folstein and McHugh, 1975); nfvPPA, patient group with nonfluent/agrammatic variant primary progressive aphasia; no., number; PIQ, performance IQ; R, right; RMT, Recognition Memory Test (Warrington, 1984); svPPA, patient group with semantic variant primary progressive aphasia; TMT, trail making test; VIQ, verbal IQ; VOSP, Visual Object and Space Perception Battery – Object Decision test (Warrington, McKenna and Orpwood, 1998); WASI, Wechsler Abbreviated Scale of Intelligence (Wechsler, 1997); y, years. A reduced number of participants completed certain tests, as follows: [a]n-1, [b]n-2, [c]n-3, [d]n-4, [e]n-5, [f]n-6, [g]n-7, [h]n-8, [i]n-9, [j]n-10, [k]n-11, [l]n-12.

Supplementary Table 7. Prevalence of reward behavioural changes in all reward behavioural clusters

Reward domain	Change	Reward-seeking		Reward-restricted		Eating-predominant		Control-like	
		n	%	n	%	n	%	n	%
Primary	Any	**12**	***100***	**9**	***75***	**39**	**95**	18	33
	Inc	***12***	***100***	***8***	***67***	***32***	***78***	*12*	*22*
	Dec	*4*	*33*	***9***	***75***	***17***	***41***	*6*	*11*
Appetite	Any	**9**	***75***	**8**	***67***	**34**	**83**	8	15
	Inc	***5***	***42***	***5***	***42***	***23***	***56***	*2*	*4*
	Dec	*4*	*33*	*3*	*25*	*11*	*27*	*6*	*11*
Sweet tooth	Any *(Inc)*	**8**	***67***	**7**	***58***	**30**	**73**	10	19
Sexual behaviour	Any	**7**	***58***	**8**	***67***	**12**	**29**	0	0
	Inc	***7***	***58***[2,3]	*0*	*0*[1]	*1*	*2*[1]	*0*	*0*
	Dec	*0*	*0*[2]	***8***	***67***[1,3]	***11***	***27***[2]	*0*	*0*
Non-primary	Any	**12**	**100**[3]	**12**	**100**[3]	**26**	**63**[1,2]	8	15
	Inc	***12***	***100***[3]	***11***	***92***[3]	***19***	***46***[1,2]	*7*	*13*
	Dec	***3***	***25***[2]	***10***	***83***[1,3]	***8***	***20***[2]	*1*	*2*
Religion	Any *(Inc)*	**8**	**67**[2,3]	1	8[1]	0	0[1]	3	6
Music	Any	**11**	**92**[3]	**8**	***67***	**17**	**41**[1]	3	6
	Inc	***11***	***92***[2,3]	*0*	*0*[1,3]	***14***	***34***[1,2]	*2*	*4*
	Dec	*0*	*0*[2]	***8***	***67***[1,3]	*3*	*7*[2]	*1*	*2*
Art	Any	**4**	***33***	**7**	**58**[3]	**8**	**20**[2]	1	2
	Inc	*1*	*8*	*2*	*17*	*3*	*7*	*1*	*2*
	Dec	***3***	***25***	***5***	***42***	***5***	***12***	*0*	*0*
Colours	Any *(Inc)*	2	17[2]	**11**	**92**[1,3]	4	10[2]	1	2

This table summarises the prevalence of altered reward behaviours in each hedonic domain for each reward cluster, as determined from the symptom survey (see text and Supplementary Table 2); raw counts and percentage of group are indicated. Significant differences (chi-square test with post-hoc $p_{FDR} < 0.05$) compared with the 'control-like' cluster are in bold; significant differences compared with other reward clusters are coded as follows: [1]significantly different from 'reward-seeking' cluster, [2]significantly different from 'reward-restricted' cluster, [3]significantly different from 'eating-predominant' cluster. Change, overall frequency and dominant direction of behavioural alteration (see main text, Supplementary Tables 2 and 3).

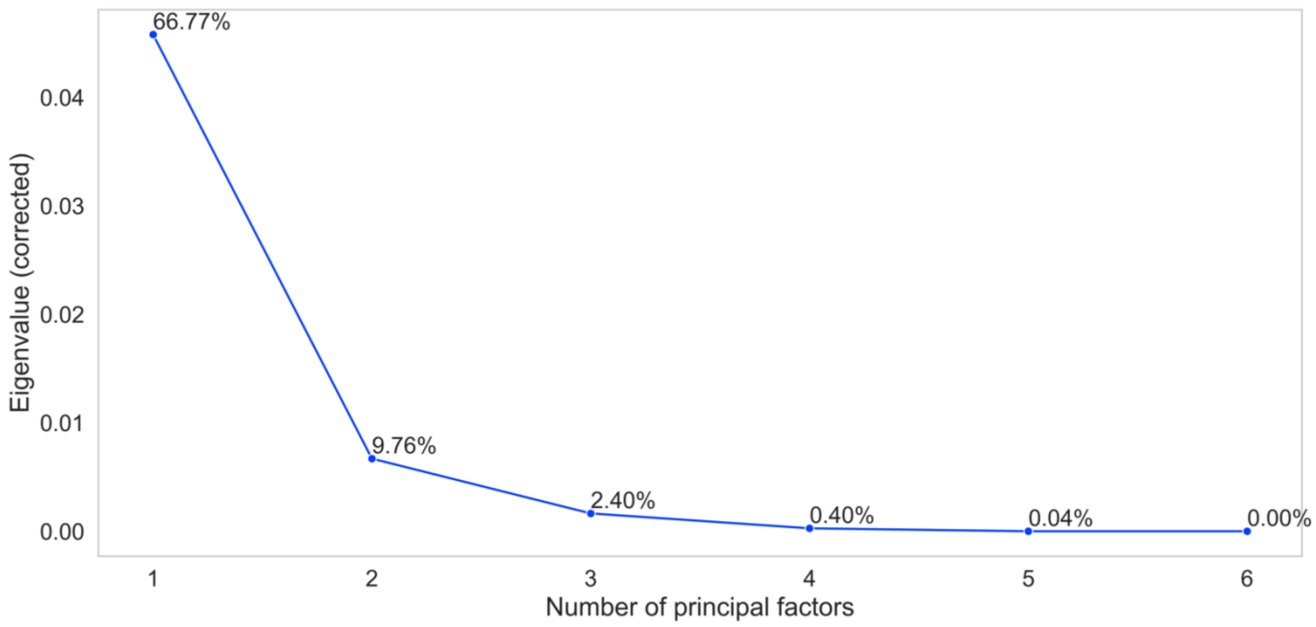

Supplementary Figure 1. A scree plot showing the eigenvalue (y-axis) of each principal factor from the multiple correspondence analysis of all reward features, after applying the Greenacre correction method. The proportion of explained variance (%) is shown for each principal factor. The ‘elbow’ in the plot trajectory indicates that most variance is accounted for by the first two principal factors.

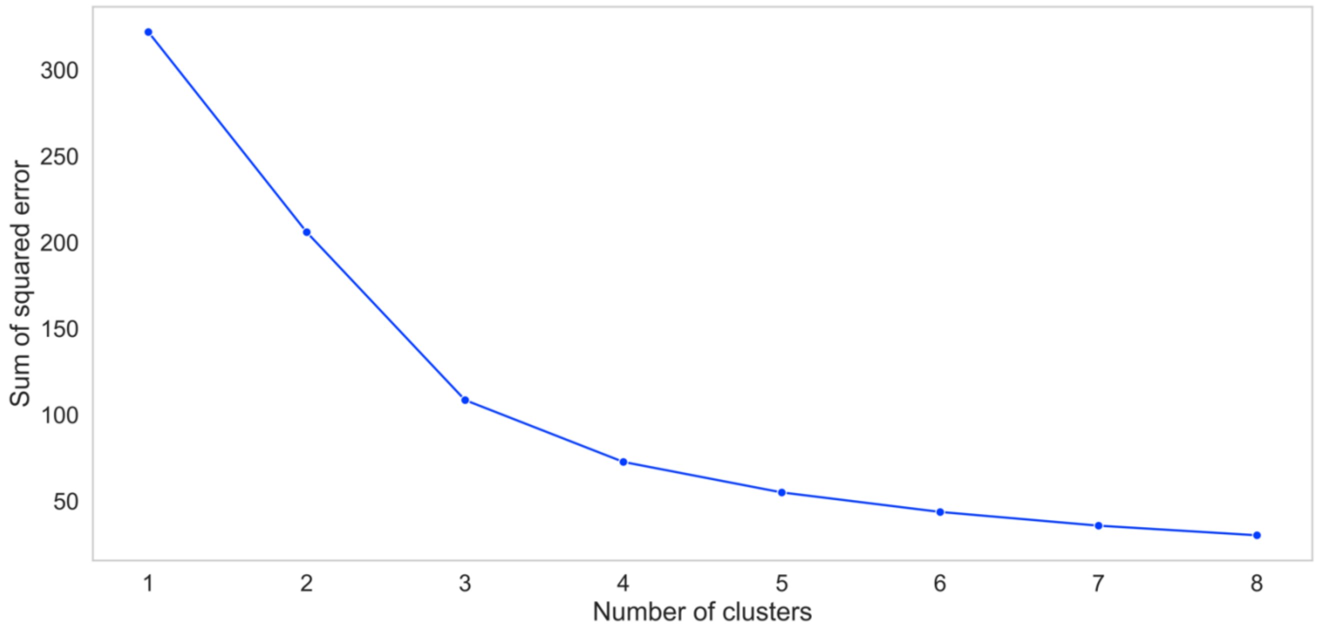

Supplementary Figure 2. A scree plot showing the value of the sum of squared errors at each number of clusters, derived from a k-means clustering algorithm on the first and second principal factors representing reward features. The ‘elbow’ in the plot trajectory indicates that most variability is accounted for by cluster n = 4.

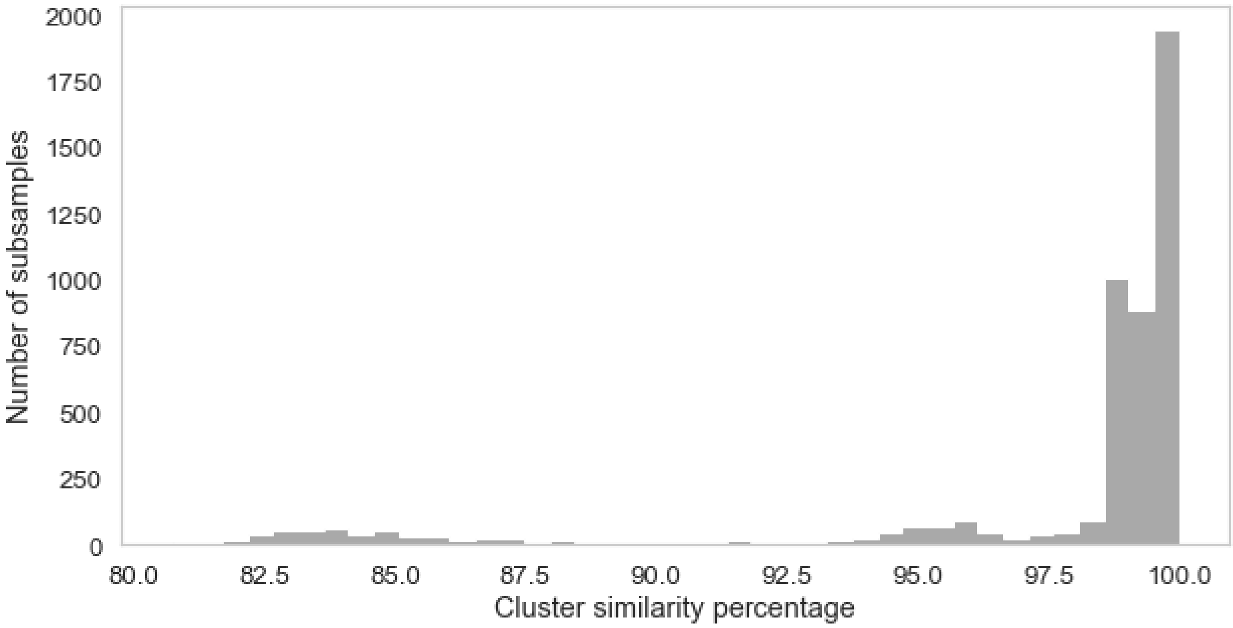

Supplementary Figure 3. A histogram showing the output of the cluster stability analysis. The x-axis shows the average percentage similarity between clusters in each subsample and the original clusters; the y-axis displays the count of the subsamples. The cluster stability analysis was performed by iteratively sampling 80% of the original data and applying the k-means clustering algorithm on the resampled dataset with replacement after each of 5000 iterations. From the graph, most iterations resulted in an average cluster similarity percentage >95%. The cluster stability index (average subsampled cluster similarity) over all 5000 iterations was 97.5%.

References

1. Ben-Hur A, Elisseeff A, Guyon I. A stability based method for discovering structure in clustered data. *Pac Symp Biocomput*. 2002:6-17.
